# Paths to Innovation in Supply Chains: The Landscape of Future Research

**DOI:** 10.1007/978-3-030-63505-3_8

**Published:** 2020-10-23

**Authors:** Rosanna Fornasiero, Irene Marchiori, Elena Pessot, Andrea Zangiacomi, Saskia Sardesai, Ana Cristina Barros, Eva Thanous, Ron Weerdmeester, Victoria Muerza

**Affiliations:** 2 STIIMA-CNR, Milano, Italy; 3 Fraunhofer IML, Dortmund, Nordrhein-Westfalen Germany; 4 INESC TEC, Porto, Portugal; 5 Aston University, Birmingham, UK; 6 Institute of Intelligent Industrial Technologies and Systems for Advanced Manufacturing, National Council of Research (STIIMA-CNR), via Alfonso Corti 12, 20133 Milan, Italy; 7 Institute of Electronics, Computer and Telecommunication Engineering, National Council of Research (IEIIT-CNR), c/o Università di Padova, via Gradenigo 6/B, 35131 Padova, Italy; 8 Fraunhofer Institute for Material Flow and Logistics, Joseph-von-Fraunhofer-Str. 2-4, 44137 Dortmund, Germany; 9 INESCT TEC Institute for Systems and Computer Engineering, Technology and Science, Campus da FEUP, Rua Dr. Roberto Frias, 4200-465 Porto, Portugal; 10 School of Engineering and Applied Science, Aston Logistics & Systems Institute, Aston University, Aston Triangle, Birmingham, B4 7ET UK; 11 PNO CONSULTANT, Avenue de la Joyeuse Entrée 1, 1040 Brussels, Belgium; 12 MIT International Logistics Program, Zaragoza Logistics Center, C/Bari 55, Edificio Náyade 5 (PLAZA), 50197 Saragossa, Spain; 13 University of Zaragoza, Quantitative Methods for Business and Economy, Gran Vía 2, 50005 Saragossa, Spain

**Keywords:** Roadmapping, Foresight, Supply chain strategies, Innovation, Resilience, Digitalisation

## Abstract

This chapter presents a Strategic Research and Innovation Agenda for supply chain and it is the result of an intensive work jointly performed involving a wide network of stakeholders from discrete manufacturing, process industry and logistics sector to put forward a vision to strengthen European Supply Chains for the next decade. The work is based on matching visions from literature and from experts with several iterations between desk research and workshops, focus groups and interviews. The result is a detailed analysis of the supply chain strategies identified as most relevant for the next years and definition of the related research and innovation topics as future developments and steps for the full implementation of the strategies, thus proposing innovative and cutting-edge actions to be implemented based on technological development and organisational change.

## Introduction

Over the next decade, supply chains (SC)s will operate more and more in an ever-changing environment, shaped by different trends (Kalaitzi et al. 2020) that strongly affect the network configuration and increase the level of uncertainty. In such a complex global context, companies need to tackle the multi-facet challenges (Gürbüz et al. 2020) raised by these trends with the support of new technologies (Stute et al. 2020) and novel SC strategies transforming the way they do business. This renovation requires not only changes in terms of assets and tools for logistics and manufacturing, but also the development of new roles and type of relationships among the actors involved in the network as well as new capabilities and competences. There is therefore the urgent need for companies to adapt the way their SCs are organised and interlinked, by transforming networks into resilient systems that are able to cope with unexpected disturbances. Coordinated actions towards the integration of manufacturing, logistics and process industries are key to strengthen economy and to reinforce the economic system for global challenges.

This chapter presents a roadmap that leads to the definition of a set of 10 SC strategies aimed at supporting industries to face future scenarios (Sardesai et al. 2020), and to the proposal of specific research and innovation paths with a medium and long term perspective, including approaches and models that exploit the potential of new technologies and collaborative mechanisms. The proposed strategies comprise a series of demanding pioneering and cutting-edge actions based on technological and organisational developments. These strategies represent a way of enhancing solid industrial skills, creativity, encouraging research and innovation and therefore they can improve the capacity of the European SCs to add value and to generate employment and wealth. With the full implementation of these strategies, European companies should invest in both, tangible (i.e. new enabling technologies) and intangible (i.e. organisational and cultural changes) assets, to face the opportunities arising from the competitive international dynamics. Transformative paths can be defined by implementing and combining one or more of the identified strategies according to the specific long-term objectives, resources and capabilities, thus supporting a continuous strive for success.

The chapter starts with a brief description of the methodology implemented to identify the research and innovation path and the steps to categorize the SC strategies according to innovation along three dimensions. The core of the chapter is from Sect. 4 to Sect. 13 where each strategy is presented including: a description of the context linked to the strategy and its most important features; a list of the main challenges of the strategy, a set of Research and Innovation Topics (RIT)s which are mapped with enabling technologies for the full implementation of the strategy and the expected impact.

## Methodology

Mapping the future of technological development is a practice adopted by all kinds of organisations (both as single company and as groups of interest and public bodies) to better anticipate new trends and forces, and their impact on different dimensions of their organisations (Boe-Lillegraven and Monterde 2015). Many types and methods for technology foresight have been developed in the last decades (Mishra et al. 2002) and technology roadmapping stands out as the most popular, being widely used to support the definition of research and innovation topics on technologies (Lee et al. 2013).

In this work, roadmapping process was chosen because it is a participatory process, involving experts in a collaborative way (Hussain et al. 2017) aiming at identifying the areas of strategic research and emerging technologies where to invest in the next years. It supports the development of knowledge about the future in the form of private and public policy making.

Therefore, for the definition of a roadmap, it is necessary to face different stages: first, it is important to outline the scope and the boundaries for the technology roadmap; then it is important to engage experts to gather the right information and the third stage comprises an analysis of the results and the data collected to consolidate the information in a more comprehensive way (Phaal and Muller 2009). In this work, taking inspiration from literature (Hussain et al. 2017), five stages have been selected: (1) Consultation with experts; (2) Clusterization of ideas; (3) Proposition of a set of SC strategies for the future; (4) Matching research themes to the SC strategies; (5) Validation.

**Fig. 1:**
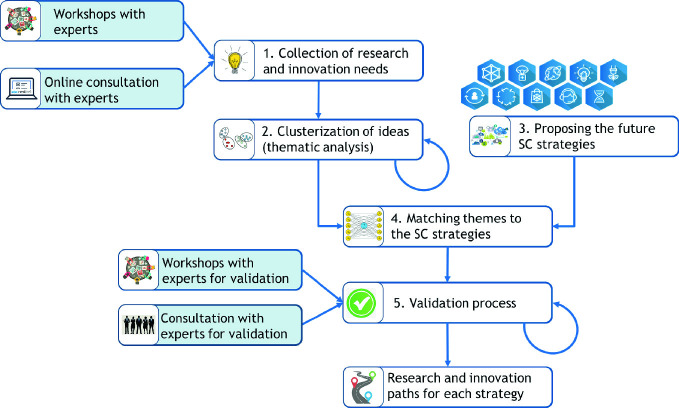
Methodology steps for SC roadmap

Figure 1 shows the methodology, described in the following paragraphs, and it highlights the steps done by the project team (white box) and the ones that required the involvement of external experts (ligh blue box). Some steps were performed in parallel during the same period and the results were combined, while other steps were structered as iterative processes since they required different back and forward interactions bebtween research team and experts in order to arrive to consistent results.

**Step 1. Collection of research and innovation needs**: experts both from academia and from industry were directly involved through two workshops and a wide consultation with a semi-structured online interview in order to collect ideas, opinions and proposals for the definition of the RITs needed to be addressed to face the SC challenges. The academics and managers invited to participate at the workshops and the online consultation represented different type of: companies (large, SMEs, start-ups), industrial sectors (i.e. automotive, steel, consumer goods, IT, fashion, transportation…), function (CEO, Operation Mangers, SC manager, industrial and academic researcher), role in the SC (i.e. managers from focal company, suppliers, vendors, outsourcers…) and research fields (SC management and configuration, manufacturing, logistics, engineering, risk management, etc.). The two workshops were organised as face-to-face brainstorming sessions in small groups of experts (in world-cafe style) each of them focusing on a particular topic under the guidance of a moderator (Krueger and Casey 2015). The interaction among participants was organised to be open-minded, with undirected atmosphere to facilitate the generation of fast-result insights (Zeng et al. 2019). Each workshop involved a sub-set of the experts according to the specific topics and aim of analysis.

The online consultation has been launched to collect other research and innovation needs based on semi-structured forms. This type of questionnaire allows to go deeper, searching for views and opinions of the interviewee and therefore to explore new paths which were not initially considered (Gray 2004, p. 217). It thus addresses issues and research areas that are important from the view of the involved experts (Kajornboon 2005). The online consultation was structured to identify:

- The most relevant specific challenges for the SCs
- SC dimensions most affected by the challenges (like manufacturing, sustainability, distribution, …)
- Enabling technologies necessary to face the highlighted challenges
- The most relevant research and innovation needs to face those challenges along the chosen SC dimension basing on experts’ experience
- The key horizontal issues related to the challenges and influencing the creation of policy recommendations (Zimmerman et al. 2020).

The experts were contacted with a formal letter of invitation where all the aims of the project were explained and timing of their involvement as well. We ended up with 100 experts involved in the period from December 2018 to June 2019.

**Step 2. Clusterisation of ideas**: thematic analysis is a qualitative research method for identifying, analyzing, organizing, describing, and reporting themes found within the collected data (Braun et al. 2006). This methodology is also useful for handling and summarizing key features of a large data set, helping to produce a clear and organised output (Nowell et al. 2017). This method aims at searching for relevant themes to describe a given phenomenon (Fereday and Muir-Cochrane 2006); this analysis supported the evaluation of 150 forms collected during the workshops and the online consultation.

The thematic analysis is supported by a review of the EU roadmaps, work programs and running projects as a means to compare the results of the consultation with existing research topics identified at European level and to avoid repetitions. These secondary resources complemented and detailed the description of the tools and approaches presented in the RITs.

In order to discover similarities between the data collected and clearly present the results of the consultation, different steps have been followed (adapted from Nowell et al. 2017 and Braun et al. 2019):

- Acquaintance with data: the identification of themes passes through careful reading of the data to familiarise with the content. The ideas were collected according to specific categories which were useful for this step to organize the data and start to separate the research and innovation needs from the horizontal issues.
- Generating initial codes: this allows to simplify and focus on specific characteristics of the data with a short phrase that symbolically assigns a summative and essence-capturing attribute to data (Saldaña 2009). Per each collected form, a short sentence was identified to summarize each specific suggestion.
- Searching for themes and review: when data has been analysed and a list of the different codes identified, it is necessary to sort and collate all the codes to extract the themes. In this step, an inductive approach was used without trying to fit codes and data into pre-existing themes or the researcher’s analytic preconceptions. The review is essential to verify if the coded data, belonging to each theme, shape a coherent pattern and, at the same time, if the themes accurately reflect the meanings which emerge from the data set. For this reason, several iterations are necessary back and forward among the researchers to arrive to a restricted list of research themes that summarize the important issues raised from experts for the identification of the research needs.

**Step 3. Proposition of a set of SC strategies for the future**: in this step, it is defined how companies should adapt their networks to face those evolutions identified in the research needs and maintain their competitiveness. A dedicated workshop was held with a restricted number of experts from academia analysing the work described in Barros et al. (2020). In fact, the following decisive SC categories were considered (Product & Service, SC Paradigm, Technology Level, Sourcing & Distribution, SC Configuration, Manufacturing Systems, Sales Channels and Sustainability) to map the features of the SCs based on the descriptions of the macro-scenarios. These characteristics were the input for the discussion during the workshop inspiring experts to identify a set of 10 strategies as the most promising to overcome the challenges of the next decade which are:

- Biointelligent Supply Chain
- Closed Loop Supply Chain
- Customer-Driven Supply Chain
- Disaster Relief Supply Chain
- Global Supply Chain
- Human centred Supply Chain
- Hyper-Connected Supply Chain
- Resource Efficient Supply Chain
- Service-Driven Supply Chain
- Urban Supply Chain.

**Fig. 2:**
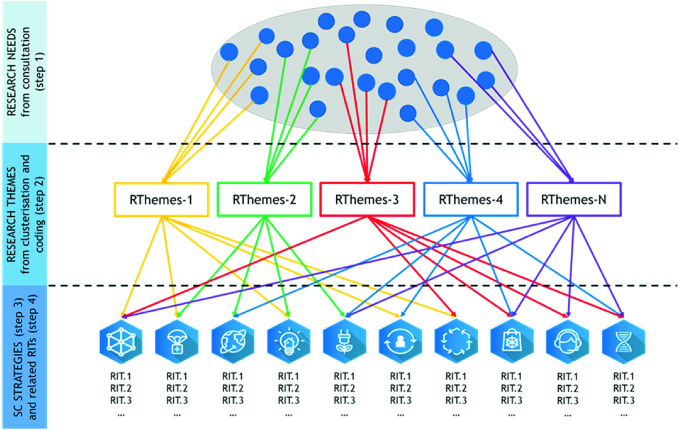
Matching research themes to the SC strategies

**Step 4. Matching research themes to the SC strategies**: the results of the step 2 and 3 were matched in order to create a coherent evolution path based on a set of RITs for each SC strategy (Fig. 2). The research themes, generated by the clusterization of forms, have been assigned to the 10 SC strategies based on their main characteristics consequently described in order to define and state the solutions and approaches needed for the full implementation of the strategies, i.e. the RITs.

**Step 5. Validation**: the RITs per each SC strategy have been defined and a validation workshop with experts was organised with the goal to review the proposing evolution paths. The feedbacks collected helped the refinement of the detailed description of each RITs. In order to reinforce the validation of these results, another iteration was done with a group of experts, who were asked to review the description of the strategies and the related RITs.

## Innovation in Supply Chain Strategies

According to Arlbjørn et al. (2011, p. 8) “SC innovation is a change (incremental or radical) within a SC network, in SC technologies, or in SC process (or a combination of these) in order to enhance new value creation for the stakeholders.” The focus on these three dimensions allows to create a path to innovation (Christiansen 2000; Mandal and Scholar 2011) as a way to gain sustainable competitive advantage relying on the competencies of many different firms within their SC network (Franks 2000; Arlbjørn and Paulraj 2013).

Other contributions available to date do not approach the concept of SC innovation from a holistic point of view and focus on investigating separately some sectors, such as for example the logistics one (Zijm et al. 2015; Lin 2008) or addressing specific issues as sustainability (Tebaldi et al. 2018), while some recent works focus on investigating the implications of I4.0 on SC management (Kagermann 2015; Hahn 2020).

In this work, the definition of innovation paths in SC was based on analysing how some specific dimensions like processes, technologies and network structure are affected taking into consideration the need of a radical change in these areas. For this reason, taking inspiration from the model proposed by Arlbjorn (2011), we define three categories where to apply innovation paths:

- Business Processes: a set of business processes have been identified and considered as object of innovation for SC: Product & Service design, Sourcing & Distribution, SC Configuration, Manufacturing Systems, Sale Channels. It is expected to have SC strategies where it is necessary to fully implement new practices and activities in specific dedicated processes with support of innovation, where it can be necessary to change the practices in some of them, or where it is necessary to fully revise well established processes.
- Technologies: this category is related to the implementation of new technologies for SCs to increase collaboration, facilitate information exchange, as well as visibility within the network (Arlbjørn and Paulraj 2013). Given our innovation-driven approach, technological changes can be considered a baseline for all the SC strategies.
- Network: it focuses on the structural aspect of the SC and how it is organised. It concerns the position of a company in the SC (i.e., distance from the end consumer) and horizontal and vertical aspects (collaboration mechanisms, partnerships, etc.). Moreover, it is related to the levels of resources used to integrate as well as manage intra- and inter-organisational processes of the SC members (Arlbjørn et al. 2011).

Given these dimensions of analysis (business processes, technologies, network) and the related degree of innovation and maturity, the following categories for the SC strategies have been proposed:

1. *Trend setting SC strategies*: this category includes novel and highly innovative strategies which are not yet applied in a wide spread manner. The implementation of these strategies implies radical changes at process, network and technological level.
2. *Advancing SC strategies*: strategies in this category are already applied but only partially spread and implemented in industry. The full implementation of these SC strategies is yet to come, because only some SC processes are on the “direction” of the strategy but not all of them have been changed or innovated. The full implementation of these SC strategies implies incremental changes at process level and radical changes at technological and network level.
3. *Revamping SC strategies*: in this category, well-established strategies are considered, and they can make a step ahead in innovation by the adoption of new digital technologies. All processes of these strategies are consolidated and well known but they can highly benefit from innovation. They imply incremental changes at process and network level and radical at technological level.

The 10 SC strategies, identified and characterised through the matching with the research themes as described in the steps of the methodology (Sect. 2), will be assigned to each of the 3 categories based on their level of innovation.

In the following sections, each SC strategy is outlined with reference to its specific challenges. The related set of RITs, derived from the technology roadmapping, are described as a way for the full implementation of innovation in the SC strategy, and the expected impacts are also reported.

## Biointelligent Supply Chain Strategy

In recent years, research in logistics has started using the phenomena of nature as a basis for creating innovative solutions and methodologies. Increasing amounts of various logistic research topics apply principles of behavioural biology, thus benefitting from the principle of nature (Tinello et al. 2017). This integrates e.g. swarm intelligence that, by now, sets the bar for several algorithms in autonomous applications. The latest research refers to Biointelligent Supply Chains (BIOSC), with a focus on mirroring nature to derive further systemic solutions and technical (digital) systems (Bio-Based Industry Consortium-Sira 2017). Future research depends greatly on interdisciplinary working groups including researchers and experts from biotechnology and engineering to SC and informatics (Fraunhofer 2019). This enables new SC structures that integrate bio-hybrid production technologies. The BIOSC strategy requires parallel concept developments of circular economy, bio-based production and digital transformation. While the latter falls short of creating a process on the way to a sustainable economy, the concepts of circular economy omit essential aspects of manufacturing industry and society (ManuFUTURE 2018). Thus, while bio-based production creates the basis, the combination of digitalisation and circular economy empower a sustainable, biointelligent economy. The aims of a BIOSC are twofold. Firstly, it follows an ecological goal in which production processes avoid harmful material and green concepts enable emission neutral SC processes. Secondly, BIOSC pursues an efficiency goal by imitating concepts from nature such as decentralised control, self-organisation and self-configuration. Biointelligent principles and systems employ nature identical and nature-analogue processes and technologies to improve production and communication for an efficient value creation along the network that should be able to restructure autonomously its configuration to achieve resilience. The aim is to design products and services to be sustainable, not to harm the ecosystem and to build an (self-)adaptable SC around those products and services. As such, biointelligent transformation is an interdependent science. It is driven by progress in various fields of science such as bio-, information and production technology.

### Specific Challenges for BIOSC

A set of specific challenges related to the features of this strategy is here reported representing the gaps to be covered with innovative approaches and tools as from the RITs in the following section.

1. *Technology Maturity*: along with the BIOSC, changes within the production processes are required, like tissue engineering and 3-D-printing. New concepts need to be developed to enhance the efficient reuse of resources. This development will progress stepwise according to the value creation by Imitation, Cooperation and Assimilation. In terms of technology, this includes production technologies to separate materials and create smart materials, e.g. based on further nano-material production flexibilities.
2. *Collaboration in a decentralised environment*: a BIOSC integrates several highly interconnected decentralised units. This includes new actors like prosumer and companies for disaggregation or amendment of products. In this decentralised environment, good collaboration mechanisms as well as new and highly adaptable organisational settings are required.
3. *Organisational Settings*: new organisational settings evolve along with BIOSC and the increasing amount of decentralised production units. Organisational structures have to adapt and to find parallels in nature.
4. *Flexible responsive SC availability*: request for production-on-demand requires a highly flexible and responsive SC. Autonomous vehicles and production units have to react flexibly to changes. The flexibility integrates a strong collaboration between SC entities to cover upcoming disturbances and to satisfy highly customised products.
5. *Resource Management in a BIOSC*: based on the concept of circular economy and the BIOSC paradigm of 100% resource efficiency, new concepts for resource management are required. This includes knowledge about availability of resources and new transportation strategies for a flexible integration of changing SC partners. New technologies and methodologies are required to enable industrial symbiosis for sharing, including sharing between small decentral units.
6. *Personalised shipments*: the idea of the DIY community and prosumers creates a high amount of personalised shipments. New ways of efficient and sustainable transportation have to be developed, including possibilities with biological assimilation and adaption of SC processes. Furthermore, an integration of autonomous vehicles along the SC needs to be implemented to incorporate and connect small decentralised units.
7. *Technological Integration for seamless connections*: to enable real-time communication, a respective interoperability for mobile devices and IoT systems, as well as communication between autonomous devices, need to be put in place. This includes the integration of heterogeneous devices and applications. Respective IT architectures are required to fulfil the interoperability and to enable a reliable stream of the increasing data flow.

### Research and Innovation Topics for BIOSC

The most important research and innovation topics for the BIOSC strategy are here described.

RIT.1: Organisational change towards a new taxonomy for biointelligent SCs

Decentralised units like self-acting autonomous transport vehicles, plants or prosumers characterise BIOSCs. Along with those decentral, autonomous and ad hoc acting entities, the roles of each actor change. The perspective of a focal company within a SC setting diverts towards a highly interconnected network of autonomously acting units, taking into consideration the multiple interfaces towards producer, customer or prosumer. Within the BIOSC, several changes are expected at production and SC level too. To guide those changes, a new and common organisational structure based on a taxonomy for BIOSCs needs to be established. Those organisational structures shall be inspired by biological settings. New matrix structures can be combined with Shared Leadership, in other cases cellular organisation or swarm organisation might be useful. As different organisational settings can apply and might get combined, the biointelligent SC requires a structural approach towards the setup of a network integrating the concept of prosumer. A new taxonomy for SCs shall support these upcoming changes and propose a way forward on how and in which way strategic goals need to be set. This work forms the basis for several organisational processes as self –organisation between decentral units. As biologists have formed their taxonomy way back, it thus can provide a primary guideline for an interdisciplinary taxonomy for BIOSCs. In this context, the model of layers systematically divides the different levels of networks, SC and production to find biological equivalents. Starting from a protozoon, logistic functional areas or systems and biological structures are compared with each other. This aims at investigating the possibilities of transferring bioequivalent processes (ten Hompel et al. 2019).

RIT.2: Nature inspired symbiotic SC models

Within this research topic, nature inspired analogies shall motivate the set-up of SCs. This requires new bio-inspired concepts to identify possibilities of increasing efficiency, to locate raw material streams and to estimate their availability. While symbiotic SC models follow the principle of lowest resistance, the concept of BIOSC builds upon a symbiosis with nature. The development towards the proposed BIOSC can be segregated best into three differently timed phases towards ‘Imitation and Cooperation with Nature’. Each phase creates a part towards a BIOSC—while imitation stands for value creation by creating an image of nature, cooperation combines biotechnology with SC structures or other forms of natural symbiosis. Imitation creates hence bio-inspired management and bio-inspired organisational settings, while cooperation stands for changing processes along with the integration of bio-based resources and bio-based materials. In the first step, the transformation includes learning from the results of nature, e.g. using sensor data for real-time decision for triggering different SC routes (see SOFiA project 2018). It implies learning from the process of evolution and from the principles of nature, e.g. in a self-organising transport model using bio-analogue optimisation methods. Conceptually, it includes the necessity for autonomous capacity and demand matching methodologies. A second phase includes imitation and cooperation and can comprise cargo ships that recoup energy through tidal forces along their journey. This symbiosis can imply material and transportation flows that are coordinated by weather (e.g. sea freight is favourable in windy weather and appropriate current). Autonomous vehicles might become independent, decentralised units that require maintenance only and their coordination become more flexible. The third phase then, involves a complex interplay of entities exchanging material and information from different sources and an adaptation of various, continuously changing SC partners. The SC consists of several decentral organised nodes within a regional area. Transport is characterised by short distances and sharing. Predictive planning supports self-structuring of the SC which can change and adjust to respective circumstances similar to a body system that reacts to physical activities. Self-organizing entities motivate their planning on the overall strategic goals of a company network. Along with the changes towards biointelligent production, a new conceptual framework for SCs is required in order to enable biointelligent SCs along the following dimensions:

1. Identifying concepts and possibilities of imitation of nature within the SC to increase efficiency.
2. Supported and inspired by the biological taxonomy, it needs to identify possibilities to realize symbioses between nature and SC. This shall lead to the development or extension of a biointelligent taxonomy for SC.
3. Identify concepts for the assimilation phase dealing with conceptual strategies for the organisation of decentralised nodes.

RIT.3: Ecosystem for biointelligent SC

In nature, SCs establish themselves via a natural bond. In a similar approach, BIOSCs and networks need to be formed. The integration of DIY partners, the concept of prosumer and the idea that each entity is consumer as well as provider of resources, results in the effect that suppliers of raw materials evolve and regress in a continuous flow. Only a highly interconnected system can ensure that new partners can be connected. A new ecosystem is required to enable a quick adaptation to new circumstances. New concepts to enable a self-structuring of SCs along with AI technologies have to be established. These concepts need to integrate not only the locating of suppliers but also the circular economy aspects to determine possible use for recycling or disaggregation of a product to reach a bio-inspired organisation. Moreover, high flexibility and quick adaptation of new ecosystems offer a great potential for the development of new business models. Continuous joining and quitting of players as well as the integration of DIY partners lead to flexible and self-structuring SC networks and to new platforms for connecting all stakeholders. The customer takes on the role of an associate producer leading to highly individualised, agile products and services. Furthermore, revenue models are continuously adapted and new (ecologically oriented) payment and pricing models arise, inevitably leading to new business models. To support these developments, a BIOSC with its decentral decision units requires different IT architectures to ensure a constant data availability with low energy demand. Next to a data exchange in real-time, effective methods for combination of data and its visualisation are required. An important aspect in this regard is short latency. Again, the concept of imitation of and cooperation with nature need to be adapted. New concepts for IT Architecture have to be established to provide immediate access to relevant data and ensure data availability between decentralised production units.

RIT.4: New SC processes to realise biointelligent SC paradigms

Along with the paradigms of BIOSCs, processes have to change accordingly. New production technologies allow for a production whenever needed. A production on demand implies material to be at hand whenever required and, therefore, it is necessary to use regional or local sources. In addition, the influence of a DIY community and the prosumer integrates several small players such as material and component suppliers or users that require re-designing processes within a BIOSC. Moreover, the advances in circular economy ensure a re-utilisation of materials. Thus, reverse logistics processes become equal to material supply processes. A new set of actors responsible for disassembling and renewal, separation and refining products, as well as other players overseeing collection and preparation are included. SC processes change, bringing with them re-utilisation and re-structuring of products and auxiliary (transport) material. This setting requires a structure of very flexible and scalable production units. Its flexibility reflects on the SC processes as raw material suppliers and related component suppliers can change on an ad hoc basis. This entails that a SC structure is not fixed but has to be agile and adapt flexibly to the requested demand and product type and find a suitable set of suppliers. With regard to the use of outputs of other production processes, the supply might need to be sourced from several entities. Further research is required in terms of new distribution systems (e.g. integration of autonomous vehicles, and integration of IoT devices or further innovation for supply mechanisms), energy and communication infrastructure (necessary to support the SC process and financial payments settings), and innovative transport systems.

RIT.5: Coordination and decision making within biointelligent SCs

Based on the concept of prosumer and the re-utilisation of any kind of output in a production process, each entity in a SC serves several customers. This evolves into a highly interconnected network of different SCs, that serve a different strategy. Accordingly, the goal within this research topic is twofold. First, concepts in nature have to be identified on how and if a common strategy for all networks exists or indeed if an external strategy has to be provided externally to achieve a bio-inspired management mechanism. Secondly, the coordination between entities and respective decision making on SC and production level has to be adapted according to bio-inspired processes. Future research has to provide concepts for decision-making, e.g. for contract negotiation, communication of capacity availability and raw material provision to allow for bio-inspired management. Each player in the production unit openly communicates its availability of resources in real-time to find additional customers. Disruptions and disturbances within a SC, like breakdowns or volcanic eruptions, trigger immediate changes of routes and suppliers on an autonomous basis. Coordination systems between autonomous entities need to have information about location of the incident and products loaded. Those examples show that new, nature based communication systems are required for the coordination within BIOSCs chains. In terms of data storage, the transfer of small packages is required. A first approach is provided by edge computing. In contrast to cloud computing, edge computing refers to decentralised data processing at the edge of a network. Data streams are processed at least partially on site (e.g. directly at the end device or on the equipment) in a resource conserving manner, but still benefit from the advantages of the cloud. Smart ecosystems support the use and reutilisation of resources—the breaking of former isolated solutions for the control of business processes and technical processes, instead forming a unified system. The flexible production environment requires traceability for raw and intermediate materials and new sharing concepts for data. Moreover, the support of connected and autonomous cars and its integration into decision making create data flows that need to be handled. Decentralised units need to be organised on a real time basis. IT Architectures, hence, have to ensure a common domain specific language and integrate new sharing concepts. Aspects of redundancy need to be considered to safeguard relevant sets of data.

### Impact

The innovation path identified with the RITs can have significant impacts on SC performance. The future impacts expected from the development of the RITs for the BIOSC strategy include:

- Increase of efficiency as an overall achievement of BIOSCs
- Increase in sustainability by ecological product life cycle management
- Increase of agility via decentralised and flexible production and transport units
- Increase in responsiveness via flexible matrix structures and open capacity communication
- Increase of reliability due to improved communication processes and common understanding
- Increase of transparency via common taxonomy.

## Closed Loop Supply Chain Strategy

Closed-Loop Supply Chains (CLSC) are networks that “include the returns processes; the manufacturer has the intention of capturing additional value and further integrating all SC activities” (Guide et al. 2003) taking back products and re-using materials or components (ALICE 2014). CLSC is strictly interconnected with circular economy, with productive systems simultaneously considering forward and reverse SC operations, starting from product design up to operational processes (Lacy and Rutqvist 2016; Webster 2015). The recycling industry, with more than 2 million informal waste pickers, is now a global business with international markets and extensive supply and transportation networks, but the potential still has to be fully exploited. As an example, in 2016 the world generated 44.7 million metric tonnes (Mt) of WEEE (Waste Electrical and Electronic Equipment), yet a mere 20% was recycled through appropriate channels (Baldé et al. 2017). Different strategies can be implemented for the management of the resources loops as proposed in Bocken et al. (2016): (i) Slowing resource loops: through the design of long-life goods and product-life extension (i.e. service loops to extend a product’s life, for instance through repair, remanufacturing); and (ii) Closing resource loops: through recycling, the loop between post-use and production is closed, resulting in a circular flow of resources. CLSCs include traditional forward SC activities as well as the additional activities of the reverse SC and the reintroduction to the market (Guide et al. 2003): (i) product acquisition from end-users; (ii) reverse logistics to move the products from the points of use to a point(s) of disposition, (iii) testing, sorting, and disposition to determine the product’s condition and the most economically attractive reuse option, (iv) refurbishing to enable the most economically attractive of the options: direct reuse, repair, remanufacture, recycle, or disposal, and (v) remarketing to create and exploit markets for refurbished goods and distribute them.

### Specific Challenges for CLSC

A set of specific challenges related to the features of this strategy is here reported representing the gaps to be covered with innovative approaches and tools as from the RITs in the following section.

1. *Strong collaboration along the closed chain*: mission business models to help manufacturers, shippers, logistic providers and users to achieve common sustainability objectives, to obtain cascading of value streams within CLSCs, to eliminate waste by designing decoupling resource use from value adding (Product-Service-Systems, Sharing etc.).
2. *Efficient resource management in return process*: capturing additional value for the return processes makes it necessary to design a more efficient (and holistic) manufacturing, collection, recovery, disposal, recycle, and reuse in all stages of the SC. Quality assurance is an important element for goods produced with recycled materials and requires transparency of materials used throughout the SC. Uncertainty of the return in terms of quantity, quality and lead time, as well as pricing, are also key challenges in CLSCs.
3. *Secure and reliable information management in industrial symbiosis*: quality of information and use of suitable channels to share information are two key elements along the SC, especially when industrial symbiosis is considered and sensitive information sharing is required to encourage companies to collaborate and join networks.
4. *Rethink regulation for Circular Economy processes*: the definition of new business models makes it necessary to rethink regulations and incentives for businesses to repair, disassemble and remanufacture products and therefore support the circular economy (Govindan and Hasanagic 2018). Take-back regulations, aimed to make manufacturers physically and financially responsible for acquiring and disposing of the used products in an environmentally friendly manner, should also be implemented.
5. *Technology development for reverse logistics, waste management and recycling, reusing and remanufacturing*: further developments of existing technologies for efficient upstreaming management of materials along the SC are needed, e.g. AI image recognition for sorting and evaluation, robotics and biotech for separation of waste. Development of existing recycling technologies further to make their input more predictable to allow better SC planning is also essential.
6. *Lack of shared KPIs for the sustainability assessment*: there is the lack of a shared criteria to evaluate the sustainability along the whole network and this makes difficult to determine who are the more sustainable actors and the impact on the environment of the SC. Criteria such as generation of solid or chemical waste, air emission, water waste disposal, other recovery options and return disposition have to be considered.

### Research and Innovation Topics for CLSC

The most important research and innovation topics for the CLSC strategy are here described.

RIT.1: Reverse logistics for recycling, reusing, remanufacturing in circular economy

Product returns in CLSCs represent a value stream, not just a waste stream and logistics is a key enabler to ensure sustainability of circular economy by providing smart and sustainable networks and services. This requires research activities related to the development of new business models, including bundled services, after-market and reverse SC, addressed with an integral approach not only in the geographical sense (urban versus rural and combined) but also for the end-to-end SC processes addressing scarce resources management. New business models and the implementation of large pilot cases should demonstrate a substantial increase of supply network efficiency and sustainability of direct and reverse flows management that are currently operated separately, but could be integrated seamlessly. To assure integration, further development and the application of technologies such as IoT and platforms will help networks to monitor goods and the information coming from the reverse flows. The use of autonomous systems and the development of big data analytics will optimise the collection of products at the end of their life integrating the forward and reverse flows. Moreover, an increasing use of smart materials and sensors will enable data collection on product usage. New tools should be developed to analyse these data and propose the best solution for recycling/ reusing/ remanufacturing goods according to the specific condition of products. Research advance is also needed to develop new sensors to identify/trace and analyse product composition. Robots and additive manufacturing technologies should ensure the efficient transformation of returning goods in valuable products for the network and external actors.

RIT.2: Industrial symbiosis and other mechanisms for collaborative SC

In order to implement closed-loop schemes, research needs to explore new mechanisms for collaboration and coordination along the SC as follows:

- Industrial symbiosis expands the existing basis of SC partnerships since it is “a form of collaborative supply chain management aiming to make industry more sustainable and achieve collective benefits based on utilisation of waste, by-products (Herczeg et al. 2018). Research should be dedicated to support companies in defining new models to reduce landfill and to transform and re-use as much waste as possible so as to close the loop and improve the use of secondary raw materials, developing new relations and business models. New ways for energy storage and usage need to be implemented, taking into consideration new demand-response approaches and sharing mechanisms. New models to share other resources like heating and water should also be developed involving not only industrial companies but also public authorities and civil society with an integrated approach; this will create an ecosystem in which environmental and business goals are combined and all the SC partners have their own responsibilities to reach sustainability. 
- New methods and tools for systemic eco-innovation approach in society towards sustainability and sharing models. Technologies such as IoT, data science and cloud-based computer systems shall be used for sharing information and resources along the SC also during the design phase, to make sustainable products to be easily recycled or remanufactured (Govindan and Hasanagic 2018) and also based on recycled materials. Eco-design is fundamental in helping companies find new solutions for the through-life management and end-of-life of products and equipment. In this sense, new tools based on collaborative platforms shall be developed to support the co-design of products in a networked environment that comply with a “make it easier to repair” approach.
- Supplier relationship management models. The increase of reused materials in the SCs has an impact on the supplier selection strategy and portfolio. It is necessary to design the structure of SC and the relationship in a more efficient and smooth way considering the trade-off between cost-efficent and environmentally friendly objectives. Research shall focus on the optimisation of the procurement infrastructure and manufacturing process, including design and development of packaging, source-to-pay, organisation and supporting IT systems.
- Customer relationship management models. new models to allow customers to receive data as a service will have to be explored to increase their awareness towards environmental and social issues. Technologies to be used to properly collect and provide added value for these data include IoT, data science, and cloud-based computer systems.

RIT.3: Digitalisation supporting closed-loop SC

Data driven SCs are characterised by full connection, integration and readiness to analyse big data. Further research is needed towards the creation of multi-actor open platforms to assure the management of the flow of information and coordinate goods and waste and related recycling processes, optimising the matching between supply and demand in the secondary raw materials market. Technologies such as IoT, data science, cloud-based computer systems, and artificial intelligence can be applied to take advantage of the use of massive and meaningful data to identify new opportunities for collaboration. Thus, research should focus on:

- Innovative managing systems: systems communication, decisions and accountability for integration of different factories, automated and distribution systems involved in recycling, reusing and remanufacturing processes.
- Integration of existing technologies and development of communication standard for horizontal integration in take back schemes and reusing processes. Establishing communication standards, practices, techniques, cost-effective, efficient means, as foundations of collaborative models for recycling processes. Foster the development and adoption of communication standards for easy data and information transfer.
- Integration of technologies and actors along the SC to understand its real time status and waste streams. Integration of edge computing in SC processes of data analysis to receive real-time data of the waste collection points.
- Integration of online marketing with operations and logistics via a digital platform to facilitate communication and information between suppliers and customers to create awareness on SC practices as a way of enhancing customer attitude towards sustainability.
- Ensuring interoperability between the various platforms used by different actors (from customers to suppliers), e.g. platforms with various waste nature (electronics, chemistry, etc.).

RIT.4: Optimisation of waste management operations

A more efficient (and holistic) design of manufacturing operations, including collection, recovery, disposal, recycling, and reuse of civil and industrial waste supported by the use of advanced automation technologies, will increase added value in the return processes of CLSCs. Research in the above-mentioned stages should include:

- Demonstrators of hub operations, transport, packaging systems, containerisation, handling technologies management, monitoring and tracing of resources throughout supply cycles for direct and reverse flows integration.
- Definition of innovative models to map relationships within and across sectorial SCs, based on identification of waste and by-product flows, barriers and opportunities for synergies in the circular economy paradigm.
- Re-design and re-planning materials, packaging, lot size, etc. using waste materials according to the specific properties and behaviour of the material itself.
- Circularity of goods and energy flows in order to achieve self-sustained triple bottom line SCs (or value co-creation in networks). Waste management from companies-symbiotic networks, including the traceability of waste flows and interoperability in circular economy.
- Zero defect production processes to eliminate waste. Studies on how to use additive manufacturing for this objective can also help. New economic frameworks to remodel/reorganize the SC in order to decrease the CO_2_ footprint for SC and/ or neutralize CO_2_ emissions.

Technologies for efficient upstreaming of materials along the SC are also needed. Automated and smart tools for sustainability should be developed to:

- Shorten the distribution network through B2C technologies for sustainable reverse logistics.
- Develop a new paradigm and rules of interaction among SC actors to fully integrate waste management flows and operations.
- Implement and adopt total cost of ownership models for new waste transportation and tracking systems.

RIT.5: Standardization of methodologies and KPIs for assessing sustainability

A diffuse increase of consciousness towards environmental issues from consumers and civil society influences the way SC activities are held. Thus, firms will have to offer environmental friendly solutions as well as demonstrate being responsible from a sustainability perspective. It will be importance to establish SC contexts consistent with the circular economy paradigm, which associate the supply and demand to improve resource efficiency. Therefore, companies need to be able to properly “assess and measure” the environmental and social sustainability level of their operations. Apart from a growing number of standards and initiatives related to sustainability, innovative and holistic measurement systems for sustainability assessment within CLSCs still have to be developed. In particular, research should be focused on:

- Defining a measurable set of indices for sustainability of SC. These can be used as a decision parameter to benchmark the sustainable development in all stages of the SC. Special emphasis should be placed on closed-loop indicators.
- Finding new ways to “certify/guarantee” sustainability of companies through evolved LCA techniques. It is necessary to make consumers aware of sustainable and socially responsible SC practices adopted by firms and understand how much more they would be willing to pay for this information.
- Using of blockchain as an enabler for the transparency and traceability along with networks, and sensor technologies to manage product information.
- Standardisation of the different methodologies and models to assess and measure the sustainability in supply cycles considering both production and logistic performance.

### Impact

The innovation path identified with the RITs can have significant impacts on SC performance. The future impacts expected from the development of the RITs for the CLSC strategy include:

- Reduction of logistics costs thanks to opportunities of synergic flows
- Reduction of Processing time (collection, sorting…) of products and waste
- Decrease of material consumption
- Increase of Energy efficiency
- Reduction of CO_2_ emissions
- Increase of use of recycled and recyclable materials.

## Customer Driven Supply Chain Strategy

The role of the consumer has been undergoing rapid changes over recent years, with an increase in the number of informed, educated, up to date consumers, in a market with a choice of products wider than ever. Social media and enterprise mobility together enable new consumption patterns also leveraged by trends, such as the middle-class explosion and the rise of individualism and personalisation. The increase in the number of middle class accounts for 64% of total consumerism, worth over $9.5 trillion (SGE 2015). Currently the middle class population spends $35 trillion annually and forecasts indicate they will spend $29 trillion more by 2030, constituting for roughly a third of projected GDP growth (in terms of purchasing power parity) (Kharas 2017). In addition, individualism emerged as a social trend demanding for a totally different customer relationship and shopping experience, offering higher level of customisation (Nielsen 2016; PwC 2017; Martinelli and Tunisini 2019). Customer expectations are on the up, and with it, a more tailored experience, and 36% of customers are interested in personalised products or services (Deloitte 2015). New production technologies such as 3D printing enable customisation and personalisation of products across multiple sectors (Ryan et al. 2017). Customers are also more willing to buy local products. Moreover, the DIY paradigm enables and encourages them to design, make or assemble products themselves. Consumers are increasingly concerned about their privacy too, and consumer protection in the digital single market became a key priority in Europe. The value of European citizens’ personal data may approach 1 trillion Euro annually by 2021 (EU 2018). Legislation on intellectual property rights (IPR) is another relevant topic affecting this strategy: eight out of ten companies that exchange data with other firms do it with their customers (83%), mainly due to their increasing involvement in product development (PwC 2018). Finally, customers prefer sustainable companies: corporate social responsibility disclosure has increased dramatically (KPMG 2017). Decision along the SC need to be driven by customer’s needs, expressing their own singularity in terms of products and services. The customer should be integrated not only as a source of capabilities but also as a co-creator developing collaboration, as it is necessary for aligning the SC from downstream to upstream by leveraging inter- and intra-organisational interactive relationships (Martinelli and Tunisini 2019). Customer driven strategies mainly rely on the agility concept, which aims for demand and production alignment, fast production and delivery of products in response to changes in customer demand (Lyons et al. 2012; Medini et al. 2019).

### Specific Challenges for CDSC

A set of specific challenges related to the features of this strategy is here reported, representing the gaps to be covered with innovative approaches and tools as from the RITs in the following section.

1. *Collaboration and Orchestration to meet customer needs*: the development of new mechanisms to enable coordination and synchronisation among all the actors of the chain to meet customer needs is required, complying with the goal of matching supply and demand and creating agile/responsive SCs.
2. *Leaner and flexible responsible SCs*: customer driven manufacturing drives a make to order (MTO) production system. This type of SC demands more intelligence and optimisation to guarantee business profitability. A flexible responsive SC through proactive procurement, JIT delivery/replenishment, and on-demand forecasting is needed.
3. *Personalised shipping drives changes in the SC*: personalised shipping increases cost of delivery/ pickup due to the increased complexity of achieving specific customers’ delivery in time and quality manner. In addition, smart management of added packaging complexity is needed, e.g. size, package, confidential information, lack of bundling opportunities).
4. *Matching custom demand*: as each customer is different, understanding customer demand is important. In this sense, the development of new models and tools for gathering and handling huge volumes of data from customers is needed. Data privacy should be taken into consideration too especially when there are several actors involved.
5. *Developing new business models*: collaborative B2B technologies are fundamental because companies have to rapidly respond to their customers’ needs while outsourcing their supply source globally (Asare et al. 2016). Explore standardization and data homogeneity along business and markets, creating open protocols and reference models (B2B, B2C and B2i) is also important.
6. *Assuring SC traceability for customer trust*: beside the alarming economic impact, when fake products enter the market, consumer safety is jeopardised, and people lose confidence in the brands they trust.
7. *Guaranteeing protected IPR*: the increased involvement of customer and supplier concerned with the development of customisation and personalisation practices and the consequent data exchange requires properly handling IRP related issues.

### Research and Innovation Topics for CDSC

The most important research and innovation topics for the CDSC strategy are here described.

RIT.1: New models and tools to understand customer needs

With the explosion in product variety and increasingly sophisticated customer needs, demand forecasting is more and more difficult. The development of new models and tools for gathering and handling a huge volume of data from customers through data science and AI represents therefore a relevant research issue. The adoption of new tools and methods for demand sensing, which will probably need to incorporate more AI and big data techniques in addition to standard forecasting methods, is essential. Models to better segment the market and novel revenue management practices will have to be explored in order to help firms in facing more diverse customer bases due to the increased product variety. Being able to charge the right price to a particular customer based on the product/service offered is not an easy task. Therefore, understanding consumer shopping behaviour, retrieving data from social media and wearable devices (using big data, machine learning, cloud computing) and how much they would be willing to pay for personalised goods is critical. Research advancements on Data Science and IoT has particular relevance, increasing the capability of learning requirements of customer preferences, habits and values, making it easier to tailor and to segment the SC to make it more efficient. Getting closer to consumers to know more about “local tastes”, understanding what drives them to ask for certain goods, to provide better service (e.g. after sales services, management of returns) is an additional crucial topic.

RIT.2: New technologies and SC models enabling personalised production

In CDSC, firms offer a wider variety of personalised products, which complicates both the product design and production processes in a distributed model. The following research issues should be addressed:

- New solutions for assuring seamless integration with product design (including the design of the final product and the input materials used in production, which requires efficient communication and coordination between second tier suppliers). Research advancements in Data science and cloud based computer systems are essential in order to develop customer data platforms (CDPs) build on first-hand data, a persistent customer base, and highly connected with current data management platforms.
- New solutions for product development and acquisition of the customer requirements together with innovative product configurators also based on VR/AR technologies, advanced measuring and configuration systems, and platforms for product monitoring.
- The development of innovative technologies for the realisation of customised products (small and lot 1 batch sizes) including smart and functionalised materials, additive manufacturing and continuous processing (e.g., 3D printing), micro-manufacturing, hybrid processes. Regarding the increased importance of DIY or prosumers, it is necessary to provide the consumers with new technology/platforms enabling their involvement in the process (also in the production process), and providing central support services (e.g., sourcing of raw materials, distributing to end consumers, collect returned goods, financial transactions when purchases occur between different entities).
- Solving IP right issues if customers are heavily involved with the “design” of the product (design right standardisation, registered designs and copyright) through advanced distributed ledger and blockchain technologies.

Furthermore, new models to allow companies to get closer to the final customers delegating customisation activities to local factories, or to customer location will have to be investigated. Future research on technologies to be able to produce in smaller facilities and new SC production/sourcing/ distribution models for this decentralised setup becomes more important. For this, containerised mobile plants, fablabs, hotspots and service centers shall be established.

RIT.3: New models and tools for dynamic Customer-Driven SC

An agile network is essential to respond to customer demand and deal with a wide variety of products, variability and personalised offer. It is thus necessary to do research on the development of innovation management systems for decentralised production models where manufacturing capabilities are spread in different facilities. This in turn, brings about the need to define new logistic systems where digitalisation can support the tracking and provision of raw materials and components, as well as supporting maintenance and remote usage of machines in real time. Solutions for automatic transformation of customer choice in SC operations shall be implemented to enable a seamless flow of information from the collection of customer requirement to its transformation in production and delivery orders. The SC has to be dynamic and automatic network configuration systems have to be explored and developed in order to enable the activation of specific SC actors, according to actual customer needs. New models and tools should be based on advances in big data analytics to increase the capacity of companies to manage large quantities of data from a variety of sources (client, suppliers, machines, and social media) and also on AI to support the selection and management of supply and distribution networks, based on real-time exchange of information between the actors involved. The use of big data along the SC supports the retrieval of customer information and the seamless transformation of customer needs for orders. The results of trends analysis from social media that support the definition of products variants and collections will have to be accessible for all SC actors and provide the customer with information and suggestions, developing an appropriate communication infrastructure. Moreover, big data can be used to activate new distributed ledger processes or similar technologies, ensuring that transferred data are original, and to conceive smart contracts for regulating different processes (from design, to production, to logistics). Tools to be developed further include the exchange of point of sales data, AI based collaborative planning coupled with forecasts, forecast replenishment, and a cross-platform data-exchange system able to facilitate the formulation of CDSC relationships. Research shall concentrate also on the development of new tools and approaches to support the definition of ad hoc SCs that need to be set time by time according to the needs of the customers, or based on agreements among themselves willing to set up new business (sharing economy). There is also the need for investigating new flexible and agile SC models that consider product modularisation strategies, postponement and “multi decoupling points” with a view to custom production and quick re-design of SCs.

### Impact

The innovation path identified with the RITs can have significant impacts on SC performance. The future impacts expected from the development of the RITs for the CDSC strategy include:

- Increase of timeliness and accuracy of data on and from customer
- Increased customer satisfaction and loyalty
- Increase of active participation of customers in design and production processes
- Reduction of time to market
- Increase of responsiveness, shorter lead times
- Reduction of the number of returns.

## Disaster Relief Supply Chain Strategy

Nowadays, modern SCs are more cross-border and integrated than ever before and they can be more vulnerable to disasters (Abe and Ye 2013). Disasters damage infrastructure and SCs, cause huge economic losses and negatively affect the global economy (Altay and Ramirez 2010). A IMF^1^’s report indicates that natural disasters such as droughts, floods, and storms can often cause damages totalling 50% of a country’s GDP (mainly for not-developed countries), with indications that frequency and size of catastrophes have risen over the past 20 years. In Europe, in the period 1980–2013, recorded losses from climate extremes cost on average EUR 11.6 billion per year (European Environmental Agency 2017) and damages are projected to increase in the future with continued climate change (Schwartz et al. 2014).

In these catastrophic scenarios, the Disaster Relief Supply Chain (DRSC) is employed. Usually this kind of strategy is adequate to provide first and immediate emergency aid as well as longer term development aid for the re-construction (Oloruntoba and Gray 2006). The DRSC can have non-profit objectives and involves logistics that account for 80% of relief operations (Van Wassenhove 2006) and are primarily reactive, being performed through ad hoc design with extensive advance planning (Balcik et al. 2010; Dubey and Gunasekaran 2016). There are three phases within disaster management that DRSC follows, namely: preparedness, immediate response and reconstruction/rehabilitation (Van Wassenhove 2006). Information management, collaboration and agility can reduce complexities (Tomasini and Van Wassenhove 2009; Oloruntoba and Gray 2006) due to uncertainties and limited knowledge about the disaster. Therefore, it is important that quick information sharing through innovative technologies and increased visibility shall be proposed to facilitate the collaboration between the many different actors (Tomasini and Van Wassenhove 2009) facilitating also partnerships between private sector, public sector and relief organisations (Balcik et al. 2010).

Given the more and more unstable conditions, the DRSC is not only useful to face humanitarian emergency, but it can also be applied by industrial networks in order to reduce the disruptive impact of uncertainties and unpredictable events on production and distribution. For example, in the last months, the pandemic caused by Covid-19 have disrupted the world affecting more than 15 millions of people globally.^2^ This emergency has forced the governments to close the boundaries and to put in lockdown the countries (such as in China, USA and most of EU countries) closing all the production and service activities. Therefore, the sanitary emergency has been followed by a deep economy crisis; OECD estimates that annual global GDP growth is projected to drop to 2.4% in 2020, decreasing around 0.5% while, due to the economic slowing down, the losses are calculated around 1 trillion $ (OECD 2020). For example in the USA more than 30 million of employees have lost their work in two months; in Europe, a report presented by ACEA (2020), has highlighted that about 1.1 million of workers have been affected by the closure of the automotive sector and the factory shutdowns have resulted in lost production amounting to 1.4 million motor vehicles in March. For companies it is important to invest into the long-term continuity of the SC, focusing also on mitigation strategies, prevention of emergency and on recovery (Fan and Stevenson 2018; Remko 2020) in order to reduce the risk of supply disruptions.

### Specific Challenges for DRSC

A set of specific challenges related to the features of this strategy is here reported, representing the gaps to be covered with innovative approaches and tools as from the RITs in the following section.

1. *Need of high collaboration level in facing emergency*: the DRSC appears to be quite fragmented with a low level of collaboration between the actors involved; these actors coming from different sectors and with different background sometimes are not able to collaborate and communicate properly. In facing quickly such events and reacting to specific needs, there is a lack of standards between the involved actors and cultural differences.
2. *Develop “leaner” and more flexible SC*: the long lead times in combination with the lack of adequate and timely information sharing and shared tools to optimise local and global solutions can make it more difficult to respond quickly to the disruptive events keeping under control the inventory levels.
3. *Identifying talents in SC during the first aid*: the shortage of talent and skills gap have created the need for specialised and trained staff able to coordinate properly the resources during emergencies.
4. *Managing risk and disruption*: as SCs become increasingly complex, risk management is vital and the network need to be able to manage disruptions and overcome rigidity and lack of reactivity towards unexpected event.
5. *Facing inventory and shipping problems*: there is poor inventory management as disasters make it difficult to store relief materials at a single place. Also, the supply system deployed in disaster relief operations depends on transportation and communication related infrastructure that are frequently destroyed by the event. Therefore, there is need to use integrated 3PL capabilities in disaster relief SCs to support NGOs and governments in responding to disasters.

### Research and Innovation Topics for DRSC

The most important research and innovation topics for the DRSC strategy are here described.

RIT.1: Multi-actor collaboration platforms for emergency

The development of multi-actor collaboration platforms can assure the management and coordination of goods and information flows to enhance collaboration between parties (Ernst et al. 2017) and inventory system. Multi-actor platforms, supported by resilient communication infrastructure, facilitate the information sharing in real time about the status of the emergency in terms of location of people in trouble, location of good and resources for first aid. Moreover, these platforms can manage donations, increase visibility and integrate all the actors involved (i.e. governments, aid agencies, private companies, donors, military, NGOs) who will get more accurate information. The use and integration of information derived from different systems can support the management of inventory and resources, transport and load consolidation. This helps to organise the delivery of goods for the first aid and the reconstruction phase, mapping countries and organisations with the right resources to ship straight away to the site of the disaster. The resources to be shared are both goods and technical staff able to support people during the emergency and organise in loco the first aid. Integration of autonomous systems is also necessary to assure delivery of goods in bad conditions. Moreover, it is essential that this kind of platform supports the collaboration with commercial SCs to organise the activities in a coordinated way. Finally, these multi-actor collaboration platforms can be used and enhanced in the preparedness phase (e.g. risk assessment, training and skills development) and not only in the response or recovery phases.

RIT.2: Crowd-help open platform for first aid

A crowd-help open platform for first aid shall enable citizens to share information in real time, support the creation of digital volunteer networks and the detection of actual needs of the affected population which, in turn, helps to mitigate the influx of unsolicited donations. This kind of platform promotes horizontal collaboration during an emergency. Citizens can be connected to the platform through their smart devices to share not only their position and instant needs, but also to receive indications on how to behave in bad situations or how to reach the nearest secure place, other information about changing environmental conditions or to share information about their health status and consequently receive indication about the behaviours to be followed. The use of mobile devices to communicate in a peer-to-peer way through collaborative platforms is essential to ensure the possibility to reach any people anywhere (Hafil et al. 2017). Moreover, this kind of platforms could facilitate the process of managing the donations, ideas and solutions as well as helping governments and NGOs to implement fast solutions not only in the immediate response but also during reconstruction. The crowd platform shall also help to train citizens and prepare them to deal with possible future emergency situations. The platform shall provide virtual courses to improve the skills of the volunteers and the professional staff who have to be prepared to support population wherever necessary. However, verification/moderation mechanisms or technologies (e.g. artificial intelligence and machine learning techniques) need to be implemented to avoid fake contents.

RIT.3: Models and tools to assure prompt response after emergency

Every disaster requires the configuration of agile/responsive SCs, planning standards in advance to overcome cultural differences. This can be enhanced by models and tools to ensure a prompt response after an emergency, such as the adoption of different technologies to accelerate the re-building phase, incorporating disaster risk reduction measures into the restoration of physical infrastructure and societal systems. It is thus important to implement:

- New technologies, including social media, big data and connected simulation tools, to develop applications for recovery that improve cooperation, communication, and collaboration. To facilitate the recovery planning process properly, appropriate and adequate resources shall be dedicated to data collection, analysis, and distribution. Such assessments are critical to enable governments affected by disasters to formulate their requests for assistance and plans post-disaster actions (UNISDR 2017).
- Technologies enabling quick response and fast re-organisation of manufacturing operations to produce the needed items for emergency in loco. The paradigm of containerised production and new 3D printing facilities could be developed (OCHA 2015).
- New optimisation systems to support inventory management, distribution networks for delivery of goods under difficult conditions.

Moreover, disaster relief standards and commitments need to be developed to assure effective assistance and common understanding. Finally, configuring the SC using local resources is also of high importance for a prompt response and reconstruction after emergency (Matopoulos et al. 2014); a beneficiary-focused, community-based approach need to be employed in the case of a post-crisis where beneficiaries become active members of the SC (Kovács et al. 2010)

RIT.4: Models and tools to handle valuable data for prevention and forecast

In adverse situations such as climate change and related disasters, humanitarian emergency, unexpected import duties, local disruptive events (i.e. plant firing with toxic emission) organisations need to be informed on time on several different dimensions that are not directly under their control, but can largely influence their operations. More specifically, models and tools that will retrieve and analyse big amount of data from different sources (both internally to the company and externally) need to be developed to handle valuable information for prevention and forecast. The application of this kind of information derived from forecasting should be linked to further innovation on the definition of tools for prompt re-configuration of SCs to make them adaptable to increased complexity and uncertainty. However, as it is extremely difficult for natural disasters to be prevented, these models and tools can help improving the humanitarian response and significantly decreasing causalities.

### Impact

The innovation path identified with the RITs can have significant impacts on SC performance. The future impacts expected from the development of the RITs for the DRSC strategy include:

- Decrease of costs of recovery plans
- Increase of completeness of information needed for decision making process
- Increase of timeliness of data sharing
- Decrease of time for emergency response and for the implementation of recovery plans
- Increase of number of people engaged in dispensing aid.

## Global Supply Chain Strategy

In 2018, the global logistics market was worth over 5.5 trillion euros,^3^ while the global supply chain (GSC) management solutions market is expected to reach $29.1 Billion by 2027.^4^ Moreover, the expansion of the GSC market is driven by the rapid increase of e-commerce industry, the change towards customised orders, the need for short delivery times, the strict standards compliance, the increasing use of location and communication. The globalisation is supported by the increase of goods and services exports seen between 2000 and 2019, 3.31% of GDP, and an increase of 3.38% on GDP on the imports of goods and services, both worldwide. The global sourcing strategy requires multiple suppliers worldwide, where companies consider procurement and purchasing across a global network, with lower costs and improved reliability, quality and access to technologies and new markets (Van Djik 2013). In the era of “time-based competition” the ability to respond rapidly to unexpected changes in demand, i.e. agility capability, is an important pre-requisit for GSC (Christopher et al. 2018). Companies should consider all sourcing options (home country as well as various near and far ones) and then decide the best for them. Reshoring trend (companies coming back to source or produce products in their home country) is also becoming a more viable option thank to enabling technologies that help companies to increase economies of scale and keep value in Europe. Looking at the downstream SC, with omni-channel sales strategy and multimodal distribution, GSCs are capable of providing decentralised distribution while holding multiple possibilities of customer-service channels in a multi-echelon system (Amirjabbari and Bhuiyan 2014; Onstein et al. 2018). Coordination and collaboration are other two important characteristics of the GSCs and trough the Physical Internet, they enable vertical and horizontal synergies for the use of resources in global networks, with significant gains in terms of efficiency and sustainability (ALICE 2014). In addition, the sustainability of the GSC is also considered with regards to distribution, where an attempt to promote “green” logistics through implementation of reverse logistics, emissions’ assessments and sustainable logistics’ procedures is consciously made (Grant et al. 2017).

### Specific Challenges for GSC

A set of specific challenges related to the features of this strategy is here reported representing the gaps to be covered with innovative approaches and tools as from the RITs in the following section.

1. *Managing global financial flows and international agreements*: need for trusting environment among SC partners, through smart contracts that positively affect financial/bill settlement strategies. Furthermore, the globalisation of SC brings forth a multitude of currencies and heterogeneous regulations, policies and taxes, all of which contribute for a complex management of agreements among SCs players, while also producing alarming levels of uncertainty. Additionally, new business models are necessary to face new complexity level, where new funding schemes can support collaborations between traditional funding agencies (such as investment banks and private equity traders) and FinTechs.
2. *Increasing sourcing and distribution complexities*: not only financial flows are affected by the constant globalisation of SC processes, rather, the operational processes that determine the global strategies are even more impacted by this change. On that note, sourcing and distribution complexity management become increasingly concerning for global companies, with special focus towards difficulties in establishing standard quality checks and procedures on different locations, resulting in less consistency in product quality control.
3. *Increasing disruptive events at global level*: risk management in global SCs becomes a major issue regarding sourcing and distribution, since there is a tendency for facing inventory and shipping related obstacles, such as the required variety of products offered on globally placed warehouses and the constant optimisation of distribution routes.
4. *Achieving seamless integration in long SC*: the combination of an increasing complexity, a lack of standardised practices of sourcing and distribution, and interoperability challenges with respect to communication on a machine-to-machine (M2M) level entails the focus point of achieving a seamless integration and synchronisation of production and logistics operations. On this aspect, the limited production capabilities, coupled with GSCs’ characteristics that require different set-ups of production facilities, may be the starting point for driving SCs’ configuration towards an overall integration of process, mostly achieved through extensive use of IoT and Communication Infrastructure.
5. *Promoting sustainable industry competition*: absence of widespread industry competition may lead to problems regarding concentration of power in multi-national companies. Thus, there is growing need for establishment of well-designed regulatory frameworks and policies to be adopted by global actors, which may require strong diplomacy efforts of reference bodies (such as governmental branches for trade agreements or specialised agencies of each industry sector).

### Research and Innovation Topics for GSC

The most important research and innovation topics for the GSC strategy are here described.

RIT.1: Global SC management with real-time optimisation and simulation

In the next years, GSC management will be affected by the full development and implementation of technologies currently applied in a jeopardised manner around the world with limited capability to integrate information and goods flows. Missing alignment in production and distribution technologies and IT infrastructure increase the difficulties with reaching SC standards. In particular, it is necessary to focus on the following research needs:

- New systems for tracking and tracing goods and services along the end-to-end SC, where the use of location technologies, smart sensors for production and packaging control comes into play. Implementation of smart contracts as a way to manage tiers of the GSC will increase responsiveness, agility and safety and security of the financial and information flows. GSC strategy needs to consider global financial flows, requiring the rapid advancement of distributed ledger technologies, all of which are still emerging and currently do not provide safe levels of cybersecurity for complex transactions.
- The use of Data Science with Big Data Analytics will also play an important role in the establishment of optimised end-to-end SCs, since the vast amount of data retrieved from sensors need to be filtered, analysed, selected and acted upon. Communication Infrastructure, IoT technologies and cloud based computer systems must be developed and implemented in a timely fashion, in order to foster the improvements needed for the aforementioned technological requirements.
- Enabling the possibility to evaluate a quick change of suppliers in case of urgent necessity. Instead of rigid SC structures, flexible SC designs have to be developed as well as its quick evaluation.
- New advanced modelling and simulation can support companies thanks to real-time data in dealing with high levels of uncertainty, multi-format distribution channels, outsourcing. Smart dashboards linked to IoT devices implemented in the production lines, logistics centres and distributers among all actors of the SC can help to quickly react to any need worldwide achieving the coordinated planning and execution of SC operations.

RIT.2: Achieving integration through seamless interoperability

In a digitalised GSC environment, the increasing complexity of sourcing and distribution processes, combined with interoperability challenges regarding machine-to-machine communication and the need for widespread information flows along the network, demand extensive research in systems integration based on seamless interoperability. The customised IT solutions entailed by GSCs actors exponentially increase this issue, since a universal solution becomes increasingly more difficult to find with the growth of the number of actors. Therefore, a substantial effort to align along the entire value chain is expected to require:

- Efficient integration of upstream and downstream processes on all nodes of the SC, implying the development of novel solutions for deeper integration which are based on modular and scalable aspects.
- Development of “plug and produce” online platforms based on reference architecture models connecting the different actors of GSCs, increasing visibility for end-users and customers while also supporting the digitisation process. This will foster B2B transactions, information flows and provide suitable simulation environments for test-bed appliances and customisation capabilities.
- Development of holistic interoperability solutions regarding the different communication protocols, with special focus on M2M and M2S, which are used on the digitalised GSC regarding smart contract establishments and material- and financial- flows.

The reference architectures used for digital industrial manufacturing, combined with AI-driven machine learning and deep learning, as well as a concise framework of standardised norms and procedures, can be achieved through extensive use of IoT-enabled devices supported by responsive and agile Communication Infrastructure and Protocols, with the necessary computational brainpower for simulated environments (SC digital twins) in real-time, on-demand requirements. This would require great use of Data Science, as well as the implementation of distributed ledger solutions that may increase the level of security of the information exchanged among SCs’ actors. The implementation of SC digital twins for the entire SC is paramount, and requires great knowledge and information management from the technical point-of-view, since it allows for real-time simulation capabilities and decision-making data storage. All of these are useful tools that may enable SC managers to look over the entire SC process, having the full transparency of information available for decision-making requirements, as well as being prompted with optimised strategic procedures from the virtualisation and simulation activities.

RIT.3: Global Shared Transportation Platforms

The geographical widespread placement of different suppliers and customers who purchase from overseas retailers through e-commerce channels, which are continuously developed offering more and more products and services, results in further complications and variables in sourcing and distribution. The advent of the Physical Internet, and its inherent consequences for global logistics providers, is a growing concern for future GSCs. Physical Internet Initiative can be considered an open global logistics system that combines physical, digital and operational interconnectivity by means of encapsulation, interfaces and universal protocols (ALICE 2014), which boasts huge advantages when relating to Global Shared Transportation Platforms (Montreuil 2011). In order to optimise the transportation channels necessary for sourcing and distribution on such large scale, the development of global shared transportation platforms with intermodal capabilities requires great efforts regarding quality procedures and assurance, as well as the effective implementation of standardised norms and processes to secure control over the increasing complexity of actors. Location Technologies and Communication Infrastructure, aided by IoT devices that connect all the transportation systems and nodes to collect data and AI methodologies are essential to facilitate the information flow of shipping procedures. These technologies will enable to realize autonomous vehicles, coupled with a fast, online, real-time AI-driven service that can gather, evaluate, decide upon information and relay actions for real-time optimisation of distribution routes, while considering intermodal possibilities that may increase the routes’ agility, safety and reliability. Another research area is the design of flexible warehouses and fulfilment centres offering postponement services and acting as innovative showrooms. Special last-mile delivery services with customised scheduling and return-to-sender capabilities are also targeted to enable global logistics to “enter” the city. Such platforms should not only be able to display the correct position and information regarding companies’ shippings, sales and purchases, but also to aid in mitigating risks related to conventional transportation methods. It is necessary to study transportation platforms connected to logistics digital twins for real-time simulation of routes, both within cities and between destinations, thus enabling companies to reach the most profitable, least risky solutions for their shipping requirements. Such a kind of digital transportation platform would enable effective track-and-trace, moving on from the current standard, which is focused on a single SC actor, to a broad overview, thus covering multiple actors on the same SC.

### Impact

The innovation path identified with the RITs can have significant impacts on SC performance. The future impacts expected from the development of the RITs for the GSC strategy include:

- Increase of timeliness of data sharing
- Increase of accuracy of data
- Reduction of transportation costs
- Reduction of time in decision making process against uncertainty
- Increase of delivery services level
- Increase of transport sustainability performance
- Increase of multimodal shipping.

## Human Centred Supply Chain Strategy

The aim of the Human Centred supply chain (HSC) strategy is the conception and development of SCs enabling the integrated and inclusive valorisation of humans, in order to contribute to employees satisfaction and well-being and to maintain humans playing a central role in production and distribution. Multiple factors are increasingly affecting quality of jobs and related skills. Highly-qualified jobs are projected to rise in the next future from 29% to 35%, while those requiring lower qualifications will fall from 21% to 15% (OECD 2014). Companies are facing disruptive changes driven by digital technologies, and certain skills are rapidly becoming obsolete while other new skills are required. For instance, software engineers, professionals in marketing, sales, manufacturing, law, accounting, and finance have to update their skills every 12–18 months (Deloitte 2017). For the next ten years, an increase in hiring SC talents with technical competencies and high level qualifications (Schröcker 2017) is expected, as well as leadership and professional competences and capabilities with cross-functional, digital skills and new talent acquisition/retention practices. Companies have to rethink the way they manage careers and deliver always-on learning and opportunities to improve workers’ skills and develop training programs to create adequate profiles for future SCs to cope with: socio-cultural talent diversity (e.g. ageing workers, cultural diversity and gender issues) and competence management (e.g. labour shortages and skill gaps), at all levels, from white to blue collars. HSC needs to take into consideration not only training but also ergonomic, safety and security: according to statistics, in 2015 in EU-28 there were in fact more than 3.2 million non-fatal accidents and almost 4 thousand fatalities as a result of occupational accidents (Eurostat 2015). To effectively address all these different issues, future SCs must become increasingly inclusive, focusing on the involvement of people, who will be enabled to perform complex activities with much added value, in a safer and more secure workplace supported by innovative devices and tools brought by digitalisation (WMF 2019). The human centred technological change in the SC processes could result not only in cost efficiency and process flexibility but also in extended corporate employee responsibility by fostering socially and inclusive responsible practices (European Commission 2014). Therefore, humans are the central element at all levels and dimensions throughout the whole SC, also implementing diversity and equity policies.

### Specific Challenges for HSC

A set of specific challenges related to the features of this strategy is here reported representing the gaps to be covered with innovative approaches and tools as from the RITs in the following section.

1. *Need of identification and development of SC skills*: while the digitalisation widens the specialised talent gaps in the EU workforce and creates talent shortage in the current marketplace, the context-aware identification of (hard and soft) SC skills is of vital importance to respond to the emerging needs of the market and better enable the sustainable management of networks. The identification of the new skills is needed to improve the effectiveness of the development of innovative learning experiences to updated the skills.
2. *Lack of training programs for workers in SC environments*: the human element in technology change management becomes important and specialised skill programmes should be designed taking into account human-centred organisational design principles to ensure the full implementation and usage of the new technologies which require advanced skills to managers, workers and drivers. The SC talent programmes, workforce development frameworks, and employee upskilling initiatives need to be re-designed to better unleash SC talent capabilities.
3. *Socio*-*cultural acceptance and awareness of future technologies*: the socio-cultural acceptance and awareness of future technologies can represent an obstacle for the successful implementation of technological change management initiatives. It is important to take into consideration the voice-of-employee when designing the future of work in SCs and to optimize technology-equipment user experience. In culturally diverse and global work settings, cross-cultural acceptance of technological enterprise solutions needs to be successfully managed. Employee upskilling interventions should take into account the socio-cultural differences in the workforce, as these differences directly affect the technology’s perceived usefulness, perceived ease of use, user attitude and user behaviour.
4. *Lack of safety in the work environment*: the integration of emerging technology solutions, such as autonomous systems and robots, with the organisational settings requires technology contingency planning and workforce risk management protocols. Furthermore, considering the environmental risks of technology solutions (e.g. toxicity levels in nanomaterial solutions), technology evaluation and monitoring processes should be re-designed in order to improve workplace health and safety conditions.
5. *Need for a social sustainable SC*: enterprises should incorporate a socially sustainable SC management approach while addressing the social issues of its SC base that are related to Occupational Health and Safety protocols and Corporate Social Responsibility protocols, among others. Furthermore, when managing the incremental digital transition in HRM and the digital transformation of SCs, making the technological systems, such as Physical Internet, socially responsible is of high importance.

### Research and Innovation Topics for HSC

The most important research and innovation topics for the HSC strategy are here described.

RIT.1: New tools to enhance work environment

This topic shall aim at providing employees with a suitable working environment. Companies, and more than ever SC activities, base operational efficiency leveraging both on high level technologies and on workers’ ability to cope with them with innovative interaction methods. In particular, collaborative robots sharing environment with humans supporting and relieving them have already been tested and are implemented in factories, warehouses and distribution centres. Further studies are necessary to improve functions and new human-machine interfaces based, for example, on visual and gestural movements for more accurate interaction with workers. Advancements are also expected in the use of exoskeletons to help workers (including the ageing workforce) in their activities, enhancing safety and productivity both in production and logistics. Research should focus on new actuators, batteries and advanced materials, as well as the development of functionalities to assure that the wearer and the exoskeleton are aligned. This research field includes the enhancement of the workplace through the design principles of ergonomics to ensure the comfort and wellbeing of employees, possibly integrating studies on: psychology to implement new analysis on human ability, capability and needs; bio-engineering to implement new tracking systems of workers’ movements; mechanical engineering to develop appropriate tools for production and distribution tasks in order to improve people’s interaction with products and systems. Finally, the use of Augmented Reality (AR) and Virtual Reality (VR) need to be developed with new low cost, ergonomic and comfortable devices, to make the interaction more natural for workers in a virtual environment, which simplify and improve tasks supplying information on processes. The devices will provide a continuous feedback of virtual objects through force feedback and will have to be auto-calibrated to easy adapt to the different operators.

RIT.2: Cyber and Physical Safety in new work environments

The flourishing of multiple human-machine interfaces and convergence of cyber and physical realms in SC management requires an integrated cyber-physical security operations management and risk mitigation approach in new workplaces to improve the safety and security of all workers involved in the networks. From the management standpoint, it is vital to protect the company’s data access controls, critical infrastructure, and eliminate any possible security threats from increased human-machine collaboration, the use of autonomous technologies and the use of smart working (increased in the last period to ensure the safety of the workers during the health crisis due to COVID-19). From the workforce standpoint, workers needs to be updated via training and certification programmes, about the operating modes of safe human-machine collaboration and integrated cyber-physical safety requirements of the new workplace environments: the workforce has to be aware about cybersecurity, privacy, and data/information due to the rapidly increasing digital footprint of the value chains (WMF 2019). Moreover, to guarantee and improve safety in the working environment, new models should be studied to solve privacy problems when tracking and tracing operators’ movements through wearable devices. New models to use data securely can help redefine the activities of the workers in case changes are needed in the SC. Considering the behavioural engineering aspects of human sensing applications, the autonomous and cognitive abilities of robots need to be improved. This means that workers and machines must be able to cooperate synergistically, sharing activities efficiently and safely. Cobots for both production and logistic processes, need to be equipped with the appropriate technology to have the sensory abilities to predict, prevent, and respond to the safety hazards in their environments. Research development of AI and the integration of new specific exteroceptive sensors (including ultrasonic, vibration and radar sensors) will improve the efficiency of prevention algorithms enabling robots to make real-time decisions autonomously and navigate within dynamic work environments. Moreover, research will have to ensure that the active exoskeletons (those that use energy sources) comply with increased human safety standards regarding the energy sources used as well as regarding the radiation emitted by electromagnetic waves from Wi-Fi, Bluetooth, etc. signals used by the various sensors. Finally, the health and the safety of the workers should be always the first priorities for companies, mainly during health crisis as in the last period, providing workers with all the safety devices, promoting the smart working and the social distancing reviewing the organisation of the tasks, form the assembly of products at the production lines to the handling of material in the warehouses or in the distribution centers and flows of people in the common spaces.

RIT.3: Technologies to identify, improve and assess workers skills

Technology will undoubtedly render certain roles in the workplace obsolete but at the same time generates a different set of roles. This is valid for digital innovation in SC management also. New methodologies to identify the skill gap and novel training programs supported by innovative learning environments should be studied, to simulate the workplace, to keep workers up to date and to help them remain competitive in the workplace also increasing investment in workforce education to reach the full potential of new technologies (WMF 2019). Digital platforms for competence management and training will support the provision of information for on-the-job training, delivering for example eLearning content to all the workers involved in SC processes or sharing the competence of human resources in order to face any possible issue adequately, both horizontally and vertically along the SC. The research challenge is to develop platforms where several different companies on the same SC can share training facilities beyond company boundaries. The development of new skills and their continuous updating, through the use of virtual training experience, need to be assessed at SC level to verify the skills attained, and manage the human resource more efficiently along the network. The paradigm of a teaching factory, already successfully implemented at manufacturing level (EFFRA 2016), can be further extended at SC level with initiatives, technologies and facilities that involve different companies in a SC to train people on production, distribution and logistics to develop the necessary skills at cross-company level. Moreover, it is necessary to study new ways of matching workers’ digital skills to each specific field of activity and job tenure, taking into consideration the need to develop soft skills, such as flexibility, creativity and teamwork capacity. Humans must be both creative and prepared to innovate in manufacturing and logistics and they need to be able to work collaboratively, virtually and remotely, along the entire network with colleagues, customers and partners alike.

RIT.4: Management of ethical issues in new models of human-machine collaboration

It is clear that in a new working environment, machines (for example robots, autonomous systems) must collaborate with humans, rather than replace them. On the one hand, they are an operationally-efficient asset, as they can help workers to handle unpredictability in their workplace. On the other hand, they have a cognitive, complex, and context specific knowledge interpretation and assessment ability that can contradict humans. Research advancement in Deep Learning should be developed, based on human needs and with the aim of expanding and augmenting human capabilities. In this way, people will gradually trust machines/technologies, seeing them as added value rather than threats to their jobs. The ethical perspective of new technologies needs be approached in two ways: people who develop computer based algorithmic systems shall be aware of possible ethical challenges, including the unintended misuse of the technology; and, when moving towards advanced autonomous systems, systems themselves shall be able to make ethical decisions to reduce the risk of undesirable behaviour. While automated decision-making systems have the potential to increase efficiency and fairness at work, they also open up the possibility of new forms of discrimination that may be difficult to identify and address. The opaque nature of Machine Learning and Deep Learning algorithms challenges our ability to understand how and why a certain decision has been made by the machine, as well as to ensure fundamental values such as equity and justice. The prejudices of people who develop a technology easily infiltrate, explicitly or implicitly, the training data of algorithms, thereby causing discrimination and injustice. Inclusion of minority groups that now remain under-represented in the areas of computer technology can help to make diversity properly represented and, consequently support to overcome discriminations.

### Impact

The innovation path identified with the RITs can have significant impacts on SC performance. The future impacts expected from the development of the RITs for the HSC strategy include:

- Decrease of risks due to incidents between workers and machines
- Increase of quality workplace
- Increase of number high skilled workers
- Increase of confidence with new technologies
- Increase of Job Quality Index
- Increase of capability to fill the skills gaps
- Increase of equity in technology usage.

## Hyper-Connected Supply Chain Strategy

Nowadays, business and society are deeply influenced by an exponential increase in connectivity that lead to the definition of a hyper-connected world. For example, thanks to Internet of Things (IoT), connected devices at worldwide level will reach 75.44 billion by 2025, up from 30.73 billion in 2020 and the market for Artificial Intelligence (AI) is expected to grow to $14.7 billion by 2025 (Accenture 2020). Digital interconnection is expanding across all industry sectors and the mastery of digital technologies in value chains offers relevant opportunities to create value for customers (Digitising European Industry—Digital Industrial Platforms 2017). Data originating within industrial contexts are considered “industrial commons” and its potential lies in so called ‘data-sharing collaboration’ between SC partners. The hyper-connected world of the future will comprise of environments transparently enriched with sensors, actuators, devices, machines, and computational elements that are interconnected and collaborating (Afsarmanesh et al. 2016) and several initiatives at EU level have been undertaken to connect factories. The full implementation of technologies such as digital platforms or decentralised exchange of information  empowers all the entities of a network to share information and the virtual elements communicate both horizontally and vertically. This transformation of the SC will enable the development of services to become more valuable, accessible and affordable. It is expected that Hyper-Connected Supply Chain (HCSC) will integrate not only continuous physical flows, but also respective information and finance flows (Büyüközkan et al. 2018); it thus allows the synchronisation of interactions between the organisations supported by intertwined digital technologies for nodes and edges integration. Once implemented, the HCSC will enable end to- end visibility and collaborative relationships through adequate data disposal. The aim of the HCSC strategy is to create a collaborative and integrated eco-system where actors from all the different levels of the SC are involved in the process of transforming data into value to improve the performance of the entire network.

### Specific Challenges for HCSC

A set of specific challenges related to the features of this strategy is here reported representing the gaps to be covered with innovative approaches and tools as from the RITs in the following section.

1. *Technology integration for seamless connection*: lack of standardised data and of a common integrated and secure IT infrastructure due to heterogeneous applications in each SC node causes mismatching of data between the nodes of the network; moreover, the lack of technology alignment at different levels of the SC generates interoperability problems and consequently it entails a low level of the quality of shared data. The technology integration problem is also strictly linked to issues of cybersecurity.
2. *Dealing with complex data environments*: each node of the chain can produce an enormous amount of data spread in many different repositories and information from the external environment has to be elaborated to face uncertainties and disturbances. It is difficult to integrate them in a SC framework enabling the seamless sharing of information to improve the performance of the entire network.
3. *Real time visibility and traceability* of the material, information and financial flows, that need to be traced in real time along the chain to avoid an untrusted and low collaborative environment. A unique source of information must also allow a complete verification of product data and finance information authenticity and quality.
4. *Role of humans in hyper*-*connected environments*: Nowadays, there is a shortage of high-skilled operators with the right skills to use new digital technologies; moreover there is a lack of trust in automation, data management and autonomous systems to be overcome.

### Research and Innovation Path for HCSC

The most important research and innovation topics for the HCSC strategy are here described.

RIT.1: Towards the implementation of a Data-Driven model

The valorisation of data along the SC is quickly evolving into an essential step for companies to build collaborative models. The objective is to encourage coordination and maintenance of information symmetry across different SC entities, with the final goal of creating agile and responsive SCs. They will become more and more dynamic: each actor generates an exponential amount of data from cyber-physical systems, industrial IoT, monitoring sensors from any tier of the chain (shop floor, distribution centre, consumer and transportation system). The full implementation of a data-driven model requires the development of new solutions to increase the quality of data, which needs to be accurate and consistent, and ensure timeliness and completeness (Hazen et al. 2014) to enable the harmonisation and the enhancement of data coming from heterogeneous sources (Viriyasitavat et al., 2019). New AI solutions, machine learning and deep learning solutions can help to extract meaningful information from multi-variant and multi-scale data, for unambiguous decisions in every day production, and delivery decision assuring quality and trust (BDVA 2018). This can also help to find unexpected patterns for the optimisation of the SC processes.

RIT.2: Platform based SC to support the creation of collaborative ecosystems

Digital platforms make data related to factories, logistics centres, and transportation providers easily accessible and shareable anytime and anywhere (ManuFUTURE 2019). Platforms allow the full integration and alignment of the actors in collaborative eco-systems and enable the creation of a SC digital twin, demonstrating the real-time status of the network to obtain an overview of the entire chain and drill down along it. Further developments are required for:

- Upstream SC platforms to integrate suppliers and outsourcers, until the alignment, and monitor the network distribution.
- Downstream SC platforms to integrate warehouses, distribution centres, and sales channels to create a stronger relationship with market and reinforce customer loyalty and involving customers during design, production, and delivery of the product.
- Horizontal platforms for human resources management to exchange information with employees on tasks, training sessions as well as monitoring safety and skills, while respecting their privacy.

The ecosystems enabled by platforms are based on the integration of different technologies and it is necessary to assure seamless and secure data-sharing for all platform users (Büyüközkan and Göçer 2018; Farahani et al. 2017) and consequently guarantee the interoperability between cloud systems and other information systems. Both open and closed platforms should be studied taking into consideration specific needs. The adoption of reference architecture, semantic technology and standardised frameworks will increase not only the interoperability but also the collaboration and trust throughout the chain.

RIT.3: Future transportation for connected SC

In recent years, progress in the development of autonomous technologies and its application in different fields like transportation and mobility has exponentially grown. However, there are still challenges to be faced like level of autonomy, complexity, safety, availability, controllability and comfort (ECS 2019). It is important to further research on how autonomous systems react in challenging and unpredictable situations (as for example unavoidable crash), analysing autonomous behaviour in increasingly complex environments (ECS 2017). The development of autonomous systems depends not only on the improvement of their functions, but also on their capability of connecting with other types of autonomous transport systems; to develop large-scale and cross-border connected systems of autonomous transport, for example drones, autonomous trucks, trains and cars. The autonomous systems involved in outbound and inbound logistic activities require an innovative and resilient communication infrastructure, an integrated network of sensors and a deep digital transformation for urban areas to guarantee optimised management of the transportation as far as the end of the chain, with the consumer in modern cities. Moreover, user awareness and acceptance of the autonomous transport system has to be increased (ERTRAC 2018).

RIT.4: Methods and approaches for the traceability and transparency of SC processes

The need of integrated track and trace solutions to ensure monitoring of products and processing of information on different levels of the network is twofold: on the one hand, it is important to ensure efficient data collection; on the other, it is necessary to implement solutions to verify the product and information along the chain. Infrastructures to collect information from different tiers of the network right through to the final consumer are necessary: new IIoT, advanced sensors and location technology will ensure monitoring products and processes, collecting data in real time. Moreover, the development of new smart products ensures continuous integration to consumers to offer new services and collect data to measure the performance until (and after) the end of its life. Decentralised systems such as a distributed ledger solution (i.e. blockchain) increases SC transparency, reinforcing credibility of the information, with real-time tracking enhancing the safety of the products along the network (Wang et al. 2019; Viriyasitavat et al. 2019) and the transparency of the financial flow. Blockchain technology ensures stored records are accurate and from a verifiable and single source (DHL 2018). It is necessary for the next years to invest in technical limitations to be overcome, such as scalability problems, computing power requirements and high energy consumption. As the perception of data value gaining importance in the global value creation, there are approaches where to invest like the one proposed in IDS^5^ which enable global dynamic data and business transactions between participants across all domains, sectors and industries without establishing a central infrastructure, thus peer-to-peer, linking single objects to entire platforms.

RIT.5: Cybersecurity to enable protection of data in SC

HCSC requires high level of trust between all the actors involved and poses significant challenges in terms of data security since the deep connection along the entire network forces each actor to exchange digital information with the outside world: a seamless flow of data and cybersecurity represent relevant challenges. The development of tools and services guaranteeing an adequate level of data security for digital collaboration in value chains is a priority. These tools need to ensure secure communication in digital transactions and data integrity during data exchange within and across the chain, and developing a stronger communication infrastructure. A system-centric view on security and privacy needs to be complemented with a data-centric view because the protection of data cannot stop at each system’s border but have to be applied over the full lifecycle of the data and therefore along the entire network (European CyberSecurity Organization 2018). The management of cybersecurity has also to provide transparency on where data resides, who has access, and for what purpose. Continuous Monitoring and Certification systems of Data Integrity and security are requested (European CyberSecurity Organization 2018). Security Service Level Agreements need to be designed at each level of the SC identifying the model and policies on how data can be used between actors without compromising business privacy. This serves another valuable purpose, i.e. maintaining good relationships with customers, who themselves are evolving naturally into more and more data sources, taking into consideration the new regulations promoted by EU Commission regarding data privacy (i.e. the new General Data Protection Regulation-GDPR).

RIT.6: New approaches to face increasing uncertainty and complexity

The coordination and configuration of the SC has to take into consideration the numerous external variables and drivers that have a strong impact on the strategic and operational decisions that companies cannot govern. These variables increase the complexity of the network and make it more difficult to optimise decisions. New models to analyse the influence of exogenous changes to SC processes more effectively are needed. The SC is similar to a complex adaptive system, because it is non-linear and dynamic, where the interconnected entities have to proactively respond to changes from both the external environment and the system itself. Although each actor operates at a different level and follows different objectives, the resilience of SC is a collective outcome (Tukamuhabwa et al. 2015); moreover, when a disruption occurs, systems has to learn to be anti-fragile: it would not lose but profit from the effects of disruption (Zitzmann 2014). Therefore, for a HCSC, it is necessary to create new tools and models to hook up the changes and consequently adapt the product, processes, production and delivery systems in a coordinated and systematic way. Principals of cognitive adaptability, self-diagnosis and self-resilience need to be put in place through the development of new tools and models supporting forecasting, production planning as well as inventory and transportation management.

### Impact

The innovation path identified with the RITs can have significant impacts on SC performance. The future impacts expected from the development of the RITs for the HCSC strategy include:

- Increase of timeliness of data elaboration
- Increase amount of data sharing for the decision making process
- Reduction of processing time
- Increase of alignment between production and logistics data
- Improvement of data accuracy
- Increase of capabilities to face cyber-attacks.

## Resource Efficient Supply Chain Strategy

Resource scarcity has become an important concern for companies, policy makers and researches as most of the traditional business models and production systems have proved to be unsustainable when it comes to the use of resources (Balatsky et al. 2015). Water scarcity has been identified as a higher risk than oil: demand for freshwater is projected to be 40% above current water supplies by 2030 (Sachidananda et al. 2016). If energy intensity remained the same over time, global energy demand would grow in lock step with GDP, almost doubling between 2017 and 2040. However, global energy demand is projected to grow only by about 20% from 2017 to 2040 because continued efficiency improvement lowers the energy intensity of the global economy (Exxonmobil 2019). In particular, industrial energy demand will raise by 50% (Exxonmobil 2016). Moreover, the volume of solid waste is expected to increase to 2.2 billion tons by 2025 (The World Bank 2012). Although the complexity of manufacturing and distributing products and services varies among different process and materials, waste is a common issue for all sectors and markets (Singh et al. 2017). Given the business and environmental costs of procurement, having a Resource Efficient Supply Chain (RESC) strategy is of crucial relevance for the successful structuration of eco-efficient cost-saving programs and green SC initiatives. While addressing all the dimensions of triple-bottom line and mutually reinforcing elements of sustainability, RESC strategy envisages the cost- and eco-efficient integration of both open- and closed-loop life-cycle systems (i.e. product design, material sourcing/selection, manufacturing processes, delivery of the final product, product return and end-of-life management of the product). The implementation of RESC strategy aims to increase the strategic compatibility, collaboration and inter-organisational awareness among SC partners. This strategy comprises four mutually inclusive, pro-environmental, and behavioural elements (Matopoulos et al. 2015): resource-aware; resource-sparing; resource-sensitive and, resource-responsive. The implementation of RESC towards circular economy can be supported by zero-waste incentives, fostering the integration of environmental management principles with industrial networks and lean and green SC strategies. In combination with the reverse logistics approach, zero-waste vision can be implemented with the harmonious blend of green procurement practices and total quality management (European Parliament STOA 2017).

### Specific Challenges for RESC

A set of specific challenges related to the features of this strategy is here reported representing the gaps to be covered with innovative approaches and tools as from the RITs in the following section.

1. *Resource efficient innovation modelling for end*-*to*-*end solutions*: need to create binding targets, raise awareness for eco-innovation, and develop frameworks to design, develop, and deploy integrated information control architecture mechanisms for a successful planning and sustainable implementation of end-to-end resource management principles using transparency via digitisation.
2. *Energy and emissions management in the manufacturing and distribution of products and technologies*: energy consumption and emissions need to be managed and reduced to the lower possible level, both in case of new and current products. A broad commitment is needed to face this challenge, including the participation of policy makers, managers, and researchers.
3. *Limitations of regulatory frameworks for successful implementation of RESC*: eco-innovation enabling regulatory cooperation mechanisms and standardised waste management protocols are needed while fostering proactive policy-to-business dialogue platforms. More specifically, environmental regulators need to engage more with other regulators in order to better assess, develop, and disseminate resource efficiency principles.
4. *Improving energy systems and diversifying eco*-*efficient energy power sources for full exploitation of digital technologies*: one of the fundamental challenges that mankind has to face in the next few years is energy supply, its storage and conversion with the lower impact possible on environment. The way humanity has developed during the last two centuries lead to unsustainable production and consumption models and, although some improvements have been achieved, much more has to be done.
5. *Limited feedstock of raw material*: the supply of some rare and scarce raw material can become difficult even more if these are the core elements of new and smart products (e.g. lithium-ion batteries for electric vehicles).

### Research and Innovation Topics for RESC

The most important research and innovation topics for the RESC strategy are here described.

RIT.1: Zero-waste production and logistics

To be efficient, the elimination of waste has to be considered at all stages of the network, requiring a broad commitment and partnership. Achieving a SC with zero waste footprint involves thus further research on several areas such as a redesign of the resource lifecycle through new tracking end-to-end systems and continuous performance assessments from procurement to packaging design and to all the production and distribution processes. It is important to reduce frictions in the intermediation of resources along the network and develop SC that are strongly programmed to reduce or eliminate waste and, at the same time, ensure minimal use of scarce environmental resources in favour of renewable energies and closed materials cycles. Moreover, a further development of methodologies, technologies and tools along the SC is expected that can support shared and integrated management of maintenance, quality control and logistics, to support zero-waste. For example, the use of IoT and the advanced analysis of the data, can model and forecast the state of degradation of the machine and the other assets at production and distribution level. These models will be able to predict deviations and the impact of a defect/waste on the subsequent SC stages and to identify proactive solutions to eliminate waste.

RIT.2: Traceability and management of product and processes information for resource efficiency

The introduction of monitoring and measurement solutions is essential in any industrial area to implement an efficient use of resources. Companies need to design, develop, and deploy an integrated control architecture for information coming from products and processes along the whole network. Related mechanisms are expected to optimise resource-efficiency of manufacturing and distribution processes by improving end-to-end traceability, creating cross-sector resource integration channels and therefore increasing SC robustness as well as the management of scarce resources. Being able to track and trace products, materials and process information in real time allows an increase in responsiveness and agility of the whole SC processes to be efficient. Further research is necessary to fully implement the adoption of smart sensors, location technologies and vision systems in production and distribution with applications like: replacing manual sampling procedures with automated and online sampling and analysis; providing important process data in real-time (temperature, flow and pressure, travel conditions); collecting data on both product and process for failure analysis, as well as on workers, drivers and operators enables defect, delay detention and product movement identification. Research efforts are required also to design and develop advanced, durable and low cost sensors that can be used where the process environment is hostile (e.g. high temperatures) or requires high resolutions of time and/or space that cannot be met by current technologies.

RIT.3: New models and technologies for resource-efficient transportation

Transportation represents a key decisional area in a RESC, especially for SC characterised by global transport, with extensive use of cross-docking areas and distribution hubs. In order to reduce transport costs, empty loads, environmental impact and new models of partnerships should be investigated. Shared transportation platforms can leverage new kinds of transportation management, thanks to better information sharing between transportation owners, logistic providers, warehouses etc. In this context, research needs to focus on the definition of models to facilitate the use of multimodal transports enabled by the combination of different transport means (i.e. trucks and train, train and vessels, trucks and drones) for which it is necessary to configure new hub networks. New methods and tools supporting the optimisation of dynamic decoupling points, cyber-sorting, lean loading and unloading operations are also necessary to increase resource-efficiency based on minimisation of journeys, use of cleaner transportation and introduction of new fuels. The alternative transport technology landscape of the EU, particularly the commercial procurement of electric vehicles (EV) powered by renewable electricity, are improving the vehicle efficiency in transport logistics towards carbon-neutral SCs. Nevertheless, there are still many open issues concerning for example: EV manufacturing technology levels; development stages of refuelling infrastructures (e.g. smart charging points); the varying country-level regulatory conditions for battery recycling (e.g. life-cycle costing) and EV charging prices. New research is necessary in order to develop transportation means that adopt Alternative Propulsion Systems, decarbonised and energy-standardised, and to study how these can be properly integrated among them and with the SC logistics and manufacturing processes. The impact of new transportation routes (like new Silk Road, or improvements to existing EU corridors) should be further studied, not only in terms of infrastructure and environment, but also with respect to the re-organisation of SC flows, location of factories, warehouses and distribution centres with models that take into consideration both private and public interests.

RIT.4: Monitoring and management of energy consumption

Energy management includes the planning and operations management of energy production and consumption activities. The following topics should be addressed:

- Identification of the origins of energy consumption in factory systems and GHG emission footprint at all stages of SC by combining manufacturing operations management with product lifecycle management, using low-cost micro-processors in electrical equipment of the factory systems, integrated in an Energy Management Systems (EMSs). This should be supported by energy cost reporting software and protocols enabling the end-to-end tracking and control of industrial energy consumption.
- Measuring the energy consumption and GHG emissions within the whole SC, and taking into account also the indirect and secondary types of consumptions and emissions (e.g. energy necessary to load trucks, and not only the necessary fuel).
- Developing novel schemas for enhancing peer-to-peer load, storage, and energy sharing mechanisms, managed on local network infrastructures of distributed energy systems and industrial IoT base of the factory systems. Development of peer-to-peer energy market platforms, where companies with demand peak can ask support to other companies in complementary ways.

Energy monitoring systems can use the IoT architecture and incorporate various technologies. In addition to real-time energy consumption information, energy monitoring systems can identify points of energy waste, or the excessive consumption of damaged devices. Data science and AI are also important technologies for the development of this strategy. Another interesting field of research concerns the worldwide distribution of scarce energy resources through intelligent SCs.

RIT.5: New approaches to energy storage

Complementary to energy consumption management, energy storage is an important component of energy efficiency and has a significant impact on SCs. The energy storage industry is constantly evolving and providing a wide range of technological approaches to manage energy supply in order to create a more resilient energy infrastructure, assure cost savings and increase sustainability both in industry and society. With the adoption of renewable energy resources, energy storage becomes even more important as these sources are often intermittent, for example producing energy only when the sun shines or the wind blows. In addition, storage technologies also improve energy quality through frequency regulation, allowing companies to produce energy when it is cheaper and more efficient and provide an uninterrupted source of energy for critical infrastructure and services. Moreover, large-scale energy storage enables the company’s electrical system to operate more efficiently, which means lower costs, emissions and more reliable energy. Given the ongoing developments of different approaches (i.e. Solid-State Batteries, Flow batteries, etc.), there is the need for further research to properly balance the investments in energy storage systems according to the SC processes and the peculiarities of each single SC. In particular, for the successful implementation of these systems, it is necessary to investigate on well-functioning, locally significant, and context aware interaction protocols, distributed coordination of SC agents, optimal energy control, and value recovery.

RIT.6: Improving data mining processes

While industry 4.0 applications are paving the way for implementing agility in manufacturing and logistics practices, industrial energy efficiency becomes a core area of concern, as the energy information becomes more scattered and distributed, owing to the co-deployment of multiple, energy consuming manufacturing tools (e.g. mobile and wearable devices, alternative propulsion systems, technologies for visual computing). In this context, intelligent storage and analysis of energy-data is needed to improve energy management decision-mechanisms at operational and strategic levels, reducing carbon intensity and electricity consumption. Moreover, even if new technologies are important allies in order to achieve resource efficiency, they can bring with them new and challenging issues. It is broadly recognised today that energy consumption in data centres are becoming a global problem. Blockchain, for example (particularly cryptographic currency mining) requires large amounts of energy consumption, creating a serious environmental problem if not properly managed. In this sense, further research is needed to allow the environmental sustainability of the inevitable grow of technological innovations.

### Impact

The innovation path identified with the RITs can have significant impacts on SC performance. The future impacts expected from the development of the RITs for the RESC strategy include:

- Decrease of waste generated in SC
- Reduction of delivery time
- Decrease of utilisation of fossil resources and CO_2_ emissions
- Reduction of energy consumption
- Increase of renewable energy utilisation
- Increase of energy storage capacity and reliability.

## Service Driven Supply Chain Strategy

Nowadays, services count for almost 75% of European GDP (Eurostat 2018), and the boundaries between physical goods and the services that companies offer are becoming increasingly blurred. Indeed, many product manufacturers are integrating services in their value proposition to raise the level of differentiation and guarantee a higher profitability and stability of revenue. The so-called “servitization” is one of the major trends characterizing recent transformations of companies’ business models across a wide range of industrial sectors. The rationales behind this global phenomenon include the opportunity of generating competitive advantage and containing costs (starting from R&D), locking in customers and increasing their satisfaction, locking out competitors with a differentiated value proposition, and also enabling environmental sustainability, by managing the complete lifecycle of assets (Giardelli et al. 2014). Servitization entails the evolution of a product-centric to a service-centric business logic that has several implications: the complexities of interactions with multiple stakeholders, the necessary change of mind-set in the collaboration with SC partners and the risk-sharing needs due to the uncertainties of the shift to the service-centric business model (Giardelli et al. 2014; Zhang and Banerji 2017). Indeed, the shift towards a Service Driven Supply Chain (SDSC) strategy requires a change from transactions to relationships, from suppliers to network partners, from elements to ecosystems (Neely et al. 2011). The SDSC aims at the establishment of an increased service business orientation and the addition of services or a focused combination of goods, services, support, self-service and knowledge building up to a service-centric SC structure. The capability of offering services as complements - or even substitutes - for available products, enabling new business models and relationships in the supply base, requires the added capability of dealing with local specific needs and partnerships with service providers. Moreover, companies traditionally linked to products manufacturing are progressively embedding digital services into physical products. Digital technologies are transforming the SC structure and the power dynamics in both downstream and upstream networks, due to reductions in production and transport costs, and the identification of different ways of engaging with customers (Vendrell-Herrero et al. 2016).

### Specific Challenges for SDSC

A set of specific challenges related to the features of this strategy is here reported representing the gaps to be covered with innovative approaches and tools as from the RITs in the following section.

1. *Developing collaborative models to support the shift to SDSC*: encouraging and accelerating trustworthy collaboration and sharing of knowledge to support the shift to service-centric business, to provide reliable services, to enhance value capture according to the new power dynamics in the SC, to successfully share resources. There is the need to conceptualize, configure or manage relationships successfully and integrate the members of the new service ecosystems (Vural 2017), especially for value co-creation.
2. *Need of workforce with expertise in services and digital technologies*: services require specialists with new types of knowledge, especially in technologies such as IoT and big data analytics, that are spreading in different industry sectors (Business Innovation Observatory 2016).
3. *Managing IP protection issues, information sharing and cybersecurity in product*-*service systems*: product firms integrating (digital) services in their offer should leverage on their unique resources, e.g. intellectual property rights or tacit knowledge, and deal with protection issues when these are outsourced, e.g. in B2B platforms, and a value co-creation perspective is considered.
4. *Dealing with drivers and implications of servitization with regard to digitalisation and sustainability*: digital technologies can be both a driver and enabler of servitization, e.g. in business model innovation towards digital servitization that mainly empower downstream companies. Moreover, services can be used as a way to reduce resources consumption and waste within the paradigm of circular economy models.

### Research and Innovation Topics for SDSC

The most important research and innovation topics for the SDSC strategy are here described.

RIT.1: Managing digital servitization of the SC

Driven by new business models and consumer habits, the so-called digital servitization encompasses technological, organisational and strategic challenges. Research advancements on AI and enhanced connectivity solutions can support the efficient analysis of data from customers and users to develop new and better product-service systems (ManuFUTURE 2019) and capitalise on them in future digitally-driven markets. There is the need to understand how the data economy paradigm affects the digitalisation of the overall SC, including the creation of collaborative models based on platforms, and their potential benefit for SCs and society as a whole. Indeed, platform-based business models such as the ones of Uber and Airbnb enable interconnections between a multiplicity of suppliers and customers. These interconnections lead to an ecosystem perspective that moves from the ownership of resources to the capability of creating better matchings between supply and demand markets, reshaping the decision-making and operations processes throughout the company and its SC boundaries. Innovative service business models enhanced by digitalisation include also services for production scheduling and machine and process optimisation, data-driven services, and Manufacturing as a Service, where manufacturing becomes fully service-oriented with small scale productions at a lower cost. While gaining efficiency in resource utilisation along single and multiple SCs, these demand-based models challenge the current manufacturing paradigm. It is necessary to study the implications for the relationship between companies in production networks, with the emergence of new SC models, and on the competitiveness of the overall SC. Moreover, further investigation is required on the interaction between IT systems, digital technologies as IoT and factory processes to sustain the ecosystem perspective.

RIT.2: Dealing with changes in business concepts and SC processes in servitized SC

The innovation in services provided to final customers, and the integration of services into product offering, result in changes to business operations (from back-office to supplies), SC structures and ways to integrate knowledge from a variety of network actors to maximise added value. Servitization of manufacturing and innovation in services can change the leading position of the focal company, foster the entrance into the market of new players with improved ability in service provision, and redefine the tiers in the overall SC. These changes can have severe impacts on:

- Power dynamics and incentives for each agent and SC tier
- Intellectual property and ownership of newly delivered solutions
- New processes and interactions between physical, information and financial flows.

Firstly, a SDSC aimed at collaboration should identify orchestration mechanisms and targeted goals for each agent in order to reach the global optimum performance for the overall SC. Secondly, studies should examine not only how servitization enables new value (co-)creation, but also how this value shifts or is shared along the SC, in terms of intellectual property and ownership. A reference model needs to be promoted for assessing organisational efforts, with defined roles and responsibilities, and related technologies, including distributed ledger, identification technologies and cloud computing for ensuring traceability and transparency on single efforts of all SC actors. Finally, further research should address the evaluation of the financial flows and payment systems of new business propositions, with specific service design methods that enhance the sharing of revenues (and long-term risks) among involved stakeholders in a continuous interaction. Indeed, a key challenge for solution providers is to reach the forecasted performance over time and in the overall SC, and configuring the bundle of services accordingly. New scopes of application of IoT and connectivity solutions, reconsidering related costs and benefits, should also be promoted to better tailor services that are acquiring a major part in the value offer, such as after-sales support and maintenance.

RIT.3: Open innovation and value co-creation for integrated product-service offer

In order to co-innovate product-service offerings in the most efficient and effective manner, SDSC should progressively evolve into new forms of collaboration. These advancements facilitate the necessary knowledge exchange and collaborative learning processes among all SC actors, starting from the involvement of customers in the value co-creation process as direct users. An investigation on the characteristics of a SC that is designed to make the most of innovation opportunities, for new product-service offers, entails several steps. These include: identifying the rationale and the available resources for co-innovation; taking advantage of available technologies (e.g. platforms) for sharing ideas and facilitating knowledge transfer; coordinating and aligning the efforts in innovation made by the different SC actors (and also other external sources); defining common policies and patterns of IPR for dealing with possible conflicts and increasing operational performance while maximizing value created. Single SC actors could act as innovation hubs, or new physical places (or virtual platforms) could be created along the SC network, where start-uppers, researchers, and companies can interact and test new ideas for product-service offerings, with the support of digital technologies and related equipment.

RIT.4: Innovative logistics paradigms and intelligent transport systems for service-driven SC

The European Commission is showing growing interest towards logistics and mobility solutions aimed at dealing with the requirements of the “on demand economy”, driven by the growth of awareness towards autonomy (and increasing importance of intelligent transport systems) and sustainability. This is in line with the key relevance of circular economy and urban manufacturing approaches, which require technological advancements beyond collaborative efforts in value chains and logistics. Specifically, the new paradigm of the “Mobility as a Service”, mainly enhanced by digitalisation, entail key opportunities to be applied to freight transportation and delivery services. Firstly, research could investigate the implications of adopting this paradigm on the structure of logistics network, the existing contracts and the business models of third party logistic providers. These could include a much more flexible system (e.g. payment of a monthly fee) for people and companies, and the value creation from different actors to benefit from transport and intralogistics services. Secondly, focusing on technological advancements, new research towards the creation of new services supporting more efficient, cost-effective logistics should consider the level of full driving automation. Future studies should focus onnovel approaches for both minimizing routes and number of freight transportation vehicles (especially in cities), and maximizing the integration between logistics, manufacturing and process industry through an enhance intelligence of delivery systems. Examples of innovative developments include the creation of large-scale, cross-border connected systems for seamless, optimised and multi-modal services for intra- and inter-logistics, and the connection between smart products and intelligent trucks directly communicating with warehouses management systems, enhanced by IoT.

RIT.5: New models and tools for secured and transparent data sharing and big data analytics in the service-centric SC

Managers and SC operators require to increase knowledge and awareness of markets, production or collaboration with partners, to face changing demand requirements in terms of services and related price. Customers themselves are becoming data suppliers and are also willing to receive data as a service, taking into account the issues of privacy and security of shared data. An extensive amount of data is also increasingly collected by sensors, IoT and identification technologies adopted to monitor both product and service processes. On the one hand, advanced tools and methods of Big Data and Analytics are considerably raising their profile and shaping the relationship with customers. Also the use of mobile and wearable devices enhances new capabilities for decision-making and delivery of services with an increasing value both for customers and providers (including upstream SC tiers). On the other hand, the ever-changing demand and shift to a SDSC are challenging single actors with vast amounts of data, and practices (and tools) for advanced data management are essential. This also entails open organisational and structural issues on data sharing, within both single organisations (e.g. between SC and marketing departments) and the overall SC (e.g. with more visibility on manufacturers and first and second-tier suppliers). New practices should be aimed at a global eco-system of data-driven services, where an end-to-end view on the supporting operations between the different actors should be ensured by e.g. adopting a distributed ledger, as well as dedicated tools for validation and security of data preserved. Collaboration setting and data/information exchange specifications have to be fixed accordingly for ensuring integrity and trustworthiness of data, systems and processes. On a prioritised basis this requires new models for cybersecurity management.

### Impact

The innovation path identified with the RITs can have significant impacts on SC performance. The future impacts expected from the development of the RITs for the SDSC strategy include:

- Reduction of waste (physical goods)
- Reduction of resources utilisation thanks to integration of manufacturing and logistics
- Reduction of stocks (assets)
- Increase of SC responsiveness to final demand
- Increase of transparency along SC
- Improvement in value offer
- Introduction of new sharing models
- Increase of number of agreements for collaborative innovation
- Reduction in lead time.

## Urban Supply Chain Strategy

The Urban Supply Chain (USC) is intrinsically related to the specific context of urban areas. Due to the rise of the urban population and the extension and multiplication of urban areas worldwide (over 68% of people will live in urban cities by 2050—United Nations 2019), the urban context becomes more predominant. Critical issues arise in the context of increased cost of living, pollution and poor air quality, traffic jam, poor food quality. In the context of SC, manufacturers and their suppliers will have to be integrated closer to their customers connected via a flexible interface. This integration is driven by the spread of new production technologies, such as additive manufacturing, and the rise of smart cities, characterised by high connectivity and new sustainable urban mobility (Manville et al. 2014; Dirks et al. 2010). Local goods, local food supplies and short circuits for delivery are in high demand (Grando et al. 2017). Due to a high level of customisation and the request of sustainability of products, the focus will be on small scale manufacturing systems in urban areas, with the growing importance of fab-labs and local producers. This kind of SC also tends to reinforce the entrepreneurship within the local community. The expansion of the urban environment involves a more complex development of the city logistics, impacting on the different flows (assets, people, vehicles etc.). With the multiplication and the intensification of these flows, it is of primary importance to optimise the last mile delivery for product components necessary for the local production process. These deliveries can be performed with autonomous vehicles and drones. The location of facilities in the city will also force companies and their logistics to be more environmental friendly and more resource efficient too.

### Specific Challenges for USC

A set of specific challenges related to the features of this strategy is here reported representing the gaps to be covered with innovative approaches and tools as from the RITs in the following section.

1. *Coping with the constantly changing urban context:* the focus is on small-scale manufacturing. Several constraints due to this specific context must be tackled and this leads to define several technological challenges. It is essential to be able to integrate the production and manufacturing into the urban web characterised by the increase of smart cities infrastructures. City logistics need to be optimised to efficiently implement last mile transportation and delivery.
2. *Optimise circulation of flows among different and inter*-*connected urban areas;* it will constitute another challenge due to issues such as traffic jam at the city entrance, overcapacity of some axis of circulation, local pollution (within port areas for instance) etc. Being able to move people and goods from one urban area to another in an efficient and sustainable way would thus be essential to promote integration.
3. *New user needs due to development and transformation of the urban web*: to understand them, it will be necessary to collect and analyze with proper tools a significant amount of data. The generated information will enable to build up and quickly reconfigure flexible networks and production lines to fully answer to the ever changing customer needs.
4. *Managing new entrepreneurial generation:* the technological transformation and the massive adoption of digitalisation will lead to a full transformation of the workforce This will impact the training and the ambition of the new generation, which will apprehend the technological transformation as an opportunity to reach their own goals and to provide a service or a product to the society. It is necessary to create an environment that can facilitate the creation of start-ups and fab-lab as a way for spread innovation.

### Research and Innovation Topics for USC

The most important research and innovation topics for the USC strategy are here described.

RIT.1: Evaluating SC impacts on urban context

Citizens, policy makers and entrepreneurs need to efficiently measure the immediate, long-term, direct and indirect impact of any new urban manufacturing implementation which will be settled within their local urban context. Due to the complexity of this kind of environment, it is essential to consider multiple perspectives (e.g. energy consumption, pollution, noise, overload etc.). Research will have to focus on the development of a new tool taking into account all the necessary KPIs linked to these different perspectives and which comply with European priorities in urban areas. Among them, it is important to consider Greenhouse Gases (GHG) emissions (direct and indirect) and other kind of pollution (rejection of pollutants, chemicals, waste generation etc.), space availability, and noise generation. All the types of data previously mentioned could be monitored and acquired via different systems. However, due to the different types of measurements, the tool must integrate several data sources spread across the city to gather real-time data and to provide different visualisation options, through a user-friendly interface. Possibility of creating different stakeholder profiles will be enabled by the tool, encompassing all kind of audiences composing the local urban context, (e.g. citizens, policy makers, entrepreneurs) to customise the use of the tool. All data have to be publicly available on a platform dedicated to the local urban context, where citizens can then take part in the discussions. The availability of all data collected and processed will lead to more informed decisions enabling each stakeholder category to accurately monitor the potential impact on the urban context. Such a solution will permit the setup of consensus among the stakeholders and decisions will be taken on real data.

RIT.2: New approaches for smart distribution in smart cities: optimisation of city logistics and of shared transportation platforms

In the urban context, distribution and last mile delivery have still to be optimised. The efficiency rate remains low and causes different issues such as lack of organisation, traffic congestion, high energy consumption, multiplication of commutes etc. In the meantime, the creation of centralised distribution centres and adapted logistics for smart cities is essential in order to implement last-mile transportation efficiently and assure distribution of materials and components. A set of different issues has to be explored like:

- City logistics and the last mile delivery trigger different issues requiring flexible solutions. Investigating the feasibility of modular systems and their implementation within the urban web should provide an alternative to the present transport system. Moreover, rail, metro and light rail systems must be envisaged as a solution to be fully exploited and the organisation of last mile delivery circuits could benefit also by using for example, automatic sorting systems directly implemented in the trucks. Collaborative solutions to manage unexpected issues (alternative itinerary in case of a road accident, maintenance anticipation, recalculation in case of a vehicle break down etc.) have also to be developed under ad hoc integration of the SC participants.
- Innovative ways of distribution (coupling trucks and drone, optimisation of delivery routes in real time, light rail systems, combining technologies etc.) in order to get a higher efficiency rate. Resource efficiency and optimisation of the loads will be among the main priorities. Another possibility to find new ways of sharing transportation and distribution is to explore how different transportation platforms are able to share their capacity. The adoption of these platforms would enable the possibility of tuning and fully optimising the existing load capacity, leading to additional delivery options.

RIT.3: Networked modular facilities and technologies for local manufacturing.

Due to automation and advancements in additive manufacturing technologies, the development and implementation of modular facilities represent an enabler for the relocation of small manufacturing sites within urban areas. In particular, building up smart and modular facilities easily and quickly adaptable to product specifications, as required by the final end users, is essential to support the product customisation and of “fast ordered-fast delivered” trends. Automation of most of the tasks can speed up order customisation. It is important to study new models for sharing these design and production facilities among different industries (for instance by using gate fees). Facilities should hence be reconfigurable and interoperable for different types of orders and products. Production lines and related logistics systems do not form one large rigid process anymore, but rather are composed of multiple modular islands, easily reconfigurable via suitable data sets available for the product requirements. The modular facilities should be adaptable to each local context, and, as far as possible with the lowest carbon footprint. The creation of new Fab-Labs will further improve the democratic adoption of technologies and their widespread use. Due to the assertion of the DIY trend, small and medium scale plants provided with manufacturing solutions can relate to service centres, to directly support the final customer in the stage of production or assembly of the specific and personalised product. Production capacities, prediction of lead times and flexibility are of first importance to deal with the expectations of end users. These modular facilities have to be easily accessible and possibly movable from one location to another. The implementation of platforms can enable an easier and shared management of these facilities both for Business to Business (B2B) and Business to Consumer (B2C) purposes. Online platforms can also support centralised sourcing for multiple DIY manufacturers in the urban area. The smart city context needs to be designed to support the development of this model and its connection to the surroundings.

RIT.4: Autonomous transportation systems in urban manufacturing and rise of awareness for their acceptability

Autonomous systems are considered as the next transport revolution for both people and goods. The adoption of autonomous transport in the urban context raises several research issues. The first regards the coexistence of autonomous and non-autonomous vehicles and public reaction towards the increasing number of autonomous vehicles in the different flows, re-characterizing the urban concept. A full study (including use of drones for delivery purposes) is required to establish an action plan for a step by step integration of autonomous vehicles within the urban context, taking into account the growing number of charging stations needed for the electric vehicles, which is closely linked to the increasing electrification of all processes. In this research, it is important to consider awareness from both perspectives: (i) people aware of sharing their space with autonomous vehicles and (ii) autonomous vehicles aware of the presence of non-autonomous vehicles around them. Fleet management processes require significant updates with the emergence of electric, autonomous and new types of vehicles. Coupling electric/autonomous trucks with drones for delivery purposes will render most of current processes obsolete. Consequences on fleet management and new methods for optimisation must be analysed and developed. Further research on autonomous light rail and autonomous metro systems have also to be considered due to their ability to move freight (not only passengers) with no Greenhouse Gasses (GHG) emission. Coordination of the different flows composing the urban context is also necessary. Analysing new modes of transport to tackle this challenge represent an option (for instance, coupling of autonomous trucks and drones for goods deliveries, aggregated modes of transportation, etc.) to be explored.

### Impact

The innovation path identified with the RITs can have significant impacts on SC performance. The future impacts expected from the development of the RITs for the USC strategy include:

- Increase of environmental sustainability and reduction of GHG emissions
- Improve of logistics agility
- Reduction of the lead times for goods and processes
- Decrease of transport time for goods and people
- Increase in the use of new transport systems
- Increase of resilience of the urban transportation
- Increase of transparency of decision making process for urban planning.

## Conclusions

This chapter presented technology roadmapping process that brought to the definition of 10 SC strategies and related RITs. Specific paths in the medium/long term are proposed in terms of contextual features, main challenges and specific lines of intervention, i.e. the RITs, with proposed solutions and enabling technologies for the full implementation of each strategy.

This work formalizes knowledge for previous studies with the support of external experts proposing a path based not only on technological development but also on new organisation models where the role of each actor in the SC is enhanced by innovative ways of collaboration. From the managerial point of view, this work proposes some paths supporting the decision-making process and each company can choose one or a combination of SC strategies taking into consideration the possible interdependency and complementarity.

In order to assign the 10 SC strategies to the three categories of innovation defined in Sect. 3 (i.e. trend setting SC, advancing SC, revamping SC), a brief description of each strategy is provided, underlining the peculiarities that influence their intrinsic level of innovation. In particular, the trend setting SC strategies, including novel and highly innovative ones not yet applied in a wide manner, imply radical changes at process, network and technological level:

- ***Biointelligent SC***. Biointelligent principles and systems employ nature-identical and nature-analogue processes and technologies to improve production and communication for an efficient value creation. The aim is to employ SC processes and services in a customizable and self-organising manner, changing the way companies network with others by imitating and assimilating processes within nature and thus to improve efficiency.
- ***Human centred SC***. Given the specific challenges arising from demographic and social trends as well as the specific needs of each categories of workers, the aim of this strategy is the conception and development of SCs enabling the inclusion of people and the valorisation of their skills, in order to contribute to employee satisfaction and well-being changing the way business processes are organised and creating new networking structures.
- ***Hyper*-*Connected SC.*** It is expected that in this SC, digitalisation is fully implemented and entities share real-time information through advanced digital platforms to communicate, monitor and manage activities thanks to connected nodes (like machines, products, factories, vehicles). All nodes are vertically integrated, as well as product lifecycle and inter-company value chain are horizontally integrated to allow the optimisation of the SC operations in smart environments.

Concerning the *advancing SC strategies* including ones already applied but only partially spread in industry due to the need to innovate some processes and to implement radical changes at technological and network level:

- ***Closed Loop SC***. It aims to integrate forward and reverse supply chain operations to support flows of product, components and other materials, such as by-products and waste. Part of the Closed-loop strategies are already implemented but waste management process to cover the entire product life cycle from cradle to grave are still in the need to further invest in research.
- ***Customer*-*driven SC***. It orchestrates every element of supply to satisfy demand wherever it occurs. Each decision along the SC has to be driven by customer’s needs, expressing their own singularity in terms of products and services. A key element is to anticipate customer demand reacting efficiently to an unstable and unpredictable demand according to an agile approach.
- ***Disaster Relief***. It is employed when disruptions (which can be either man-made or natural) affect society and business, threatening its objectives and needs, and impact on companies’ operations. To face the first phases of the disasters, the SC operations presents non-profit objectives and NGOs, Governments and companies work together to ensure a prompt response for the first aid and then help the population in the reconstruction. In this complex environment, companies need to develop strategies to recover and prevent uncertainties and disruptions.
- ***Resource Efficient SC***. It aims to deliver greater value with less input thus reducing environmental impacts. This model contributes to the increase awareness capacity of SC partners in eco-efficient operations management practices at downstream level with the right scale of low-carbon disclosure mechanisms, ethical, responsible sourcing activities, supplier contracting and purchasing decisions at upstream level.
- ***Service*-*driven SC***. It entails the evolution from a product-centric to a service-centric business logic and SC, innovating from transactions to relationships, from suppliers to network partners, from elements to ecosystems. It is aimed at the establishment of an increased service orientation and the addition of services or a focused combination of goods, services, support, self-service and knowledge until reaching a service-centric structure.
- ***Urban SC***. The urban areas are characterised by high level of development, high density of people, a concentration of human structure (such as houses, commercial buildings, bridges, roads, railway), wealth, goods and services. In that prospective, Urban SC can deal mainly with the DIY paradigm and personalised production/delivery requiring local and glocal sourcing, personalised shipping. Changes in the networking structures expecially with customers and society is necessary to be put in place.

As for the *revamping SC strategies*, with well established approach where all processes and networking systems are consolidated and well known but can benefit from a radical change at technological level:

- ***Global SC***. In the global SC product and services are exchanged in a dynamic worldwide network; the multiple suppliers of raw materials and components, the decentralised manufacturing and the multimodal distribution increase the complexity of information-, material- and financial flows.

It emerged that SCs and their evolutions should be intended as complex network structures that interact with their context and the other SCs through relationships that affect (and are affected) by their strategic choices. Companies should then understand the proper combinations of the SC strategies, adapt them according to their contextual features, and self-organize in the dynamic environment in order to remain competitive. The development of specific RITs and related solutions should be targeted and complemented according to the priorities in terms of performance and impacts. Further efforts should be done to create a structured system of assessment to help companies to evaluate the best strategies for their specific case and the needed capability to implement each RIT.

## Footnotes

1 https://www.imf.org/external/pubs/ft/fandd/2019/12/pdf/fd1219.pdf.

2 https://covid19.who.int/?gclid=Cj0KCQjw6uT4BRD5ARIsADwJQ1-xZINZeB9iaMD4zUYl8rwgWFYqpWSgEurk8Nq8HRfR2AKDnELZ7rAaApFcEALw_wcB (accessed on 23 July 2020).

3 Statista, 2020. https://www.statista.com/statistics/1069868/total-global-logistics-market-size-region/#statisticContainer.

4 https://www.businesswire.com/news/home/20201013005874/en/29.1-Billion-Supply-Chain-Management-Solutions-Market---Global-Trajectory-Analytics-to-2027---ResearchAndMarkets.com.

5 https://www.internationaldataspaces.org/our-approach/.
